# Integrative network biology analysis identifies miR-508-3p as the determinant for the mesenchymal identity and a strong prognostic biomarker of ovarian cancer

**DOI:** 10.1038/s41388-018-0577-5

**Published:** 2018-11-26

**Authors:** Linjie Zhao, Wei Wang, Lian Xu, Tao Yi, Xia Zhao, Yuquan Wei, Louis Vermeulen, Ajay Goel, Shengtao Zhou, Xin Wang

**Affiliations:** 1 Department of Obstetrics and Gynecology, Key Laboratory of Birth Defects and Related Diseases of Women and Children of MOE and State Key Laboratory of Biotherapy, West China Second University Hospital, Sichuan University and Collaborative Innovation Center, Chengdu, China; 2 Department of Biomedical Sciences, City University of Hong Kong, Kowloon Tong, Hong Kong; 3 Department of Pathology, West China Second University Hospital, Sichuan University, Chengdu, China; 4 Laboratory for Experimental Oncology and Radiobiology (LEXOR), Center for Experimental Molecular Medicine (CEMM), Academic Medical Center (AMC), University of Amsterdam, 1105 AZ Amsterdam, The Netherlands; 5 Center for Gastrointestinal Research and Center for Translational Genomics and Oncology, Baylor Scott and White Research Institute and Charles A. Sammons Cancer Center, Baylor University Medical Center, Dallas, TX USA; 6 Shenzhen Research Institute, City University of Hong Kong, Shenzhen, China

**Keywords:** Ovarian cancer, Prognostic markers

## Abstract

Ovarian cancer is a heterogeneous malignancy that poses tremendous clinical challenge. Based on unsupervised classification of whole-genome gene expression profiles, four molecular subtypes of ovarian cancer were recently identified. However, single-driver molecular events specific to these subtypes have not been clearly elucidated. We aim to characterize the regulatory mechanisms underlying the poor prognosis mesenchymal subtype of ovarian cancer using a systems biology approach, involving a variety of molecular modalities including gene and microRNA expression profiles. miR-508-3p emerged as the most powerful determinant that regulates a cascade of dysregulated genes in the mesenchymal subtype, including core genes involved in epithelial–mesenchymal transition (EMT) program. Moreover, miR-508-3p down-regulation, due to promoter hypermethylation, was directly correlated with metastatic behaviors in vitro and in vivo. Taken together, our multidimensional network analysis identified miR-508-3p as a master regulator that defines the mesenchymal subtype and provides a novel prognostic biomarker to improve management of this disease.

## Introduction

Epithelial ovarian cancer ranks the top most deadly gynecologic cancer worldwide [1]. The majority of histological ovarian cancer types belong to high grade serous ovarian carcinoma (HGSOC), with relatively poor prognosis due to the prevalently late stage of disease at diagnosis, widespread dissemination and a high relapse rate. Early stage HGSOC patients could achieve up to 90% cure rates with current therapies, but these rates drop remarkably in late stage patients [2]. In contrast to other cancer cell types, ovarian cancers could metastasize by spreading to nearby organs or by dissemintate throughout the peritoneal cavity. Due to the diffuse nature of ovarian cancer, the current standard therapeutic strategy for ovarian cancer is resection of the primary tumor combined with adjuvant chemotherapy. Although most ovarian cancer patients initially respond to such therapeutic regimen, most ultimately die of recurrence [3]. Therefore, further exploration of the molecular mechanisms underlying ovarian cancer pathogenesis and progression are urgently in need.

With notable heterogeneity, ovarian cancer, until recently, has only been reflected by histopathological categorization and mutation status of major cancer genes [4]. Nevertheless, the molecular diversity of this deadly disease renders it hard to precisely classify and treat. Only lately, Tothill et al. employed unsupervised classification of gene expression patterns from 285 ovarian cancer patients, identifying four transcriptionally different ovarian cancer subtypes: immunoreactive, differentiated, proliferative, and mesenchymal subgroups. This study further demonstrated that the mesenchymal subtype had relatively poor overall survival [5]. More recently, the mesenchymal subtype was recapitulated by the Cancer Genome Atlas (TCGA) network using RNA-sequencing data for about 500 serous ovarian cancer patients. Therefore, the quest for identification of the driving molecular events underlying the poor prognosis mesenchymal subtype is indispensable for a more precise targeting of ovarian cancer patients.

MicroRNAs are short non-coding RNAs that orchestrate mRNA translation into proteins [6]. Individual microRNAs can affect many genes simultaneously, underscoring their influence on the expression of complete networks determining a specific cancer subtype. In fact, accumulating evidence has implicated that microRNAs can drive poor prognosis subtypes by regulating the expression of genes actively involved in aggressive cancerous features including the epithelial–mesenchymal transition (EMT) phenotype, a process in which loss of epithelial hallmarks and acquisition of mesenchymal features can be observed in cancer cells, enabling them to metastasize to distant organs [7, 8]. Accumulating evidence implicates that acquisition of invasiveness in ovarian cancer cells is accompanied by EMT [9]. In this study, we demonstrate a network-based systems biology strategy that facilitates the discovery of subtype-specific drivers for different ovarian cancer subtypes. The dominant network identified in the mesenchymal group is master regulated by miR-508-3p. miR-508-3p promoter region methylation regulates the activity of this network and manipulates gene expression of the poor prognosis subtype of ovarian cancer. Thus, we showed that in ovarian cancer, miR-508-3p not only determines distinct cellular states, but also regulates subtype-specific gene expression, through manifestation of mesenchymal ovarian cancer subtype. Defining the regulatory network of mesenchymal tumors allows us to identify miR-508-3p promoter methylation as a molecular marker that can be used to determine subtype category of ovarian cancer at early stages, but more importantly laid a theoretical foundation for precision therapy of ovarian cancer patients.

## Results

### Transcriptomics profiling defines a heterogeneous subgroup of poor prognosis ovarian cancer

Ovarian cancer is a heterogenous disease, which could be divided into four major subgroups, immunoreactive, differentiated, proliferative and mesenchymal subtypes [5, 10]. Recently, using Bonome cohort (*n* = 182) as the training dataset [11], Konecny et al. recapitulated the four molecular subtypes defined by TCGA dataset, of which the mesenchymal subtype was found to be significantly associated with poor survival [12] (Supplementary Fig. S1a). In an effort to identify the molecular determinant that may specifically drive the mesenchymal subtype of ovarian cancer, we analyzed gene expression profiles from more than 1000 patient specimens from the public cohorts, followed by in-house validation in the West China cohort (*n* = 131) (Supplementary Table S1). Using the Bonome cohort as our training set, we built a 10-gene classifier based on PAM (prediction analysis for microarrays) algorithm [13] to distinguish the mesenchymal subtype from the others (Supplementary Table S2). As expected, we indeed observed patients classified into the mesenchymal subtype had concomitant poor survival (Supplementary Fig. S1a), which was further confirmed in the Tothill (*n* = 285) and Mateescu cohort (*n* = 107) (Supplementary Fig. S1b, c and Supplementary Table S3).

### A microRNA regulatory network fine-tunes differential gene expression between mesenchymal and non-mesenchymal ovarian cancer

**Fig. 1:**
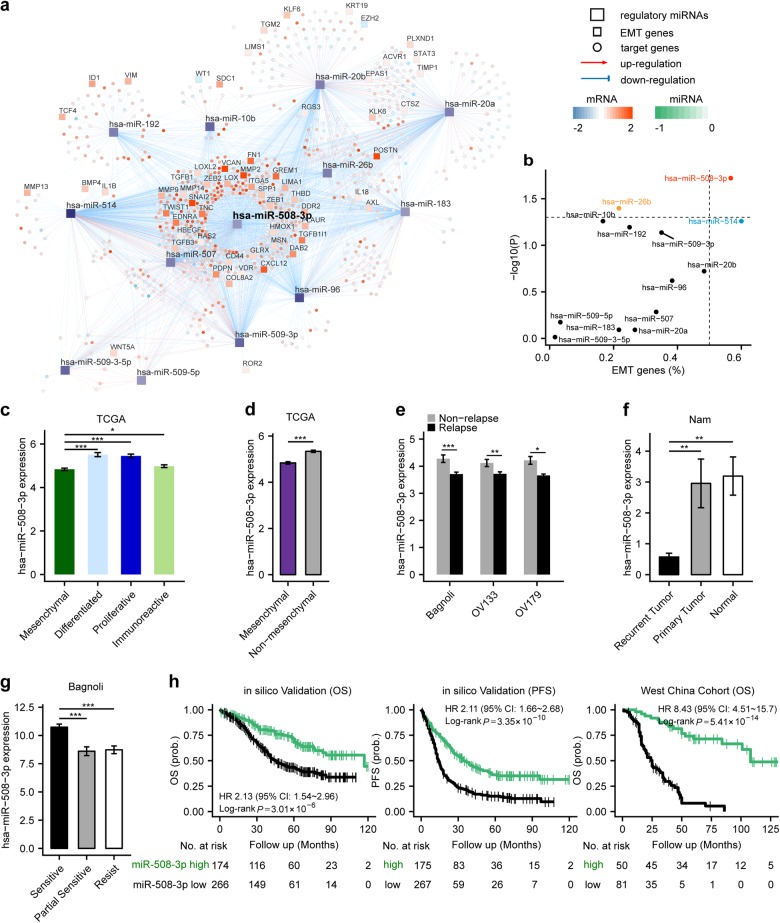
Network biology analysis defines miR-508-3p as the major regulatory network of mesenchymal-specific and EMT genes. **a** The differential microRNA expression between mesenchymal and non-mesenchymal ovarian cancer in the TCGA data set (green: lowly expressed in the mesenchymal subtype). Predicted genes regulated by the microRNAs are shown according to their differential expression between mesenchymal and non-mesenchymal tumors (blue: lowly expressed in the mesenchymal subtype, orange: highly expressed in the mesenchymal subtype). The edges between microRNAs and genes are depicted in red (induction) or blue (repression) based on the predicted signs of regulations of miRs on genes. EMT genes from the Taube EMT signature [15] are underscored as rectangles. **b** Statistical significance of overrepresentation of a microRNA’s regulon for EMT genes (−log10 transformed Benjamini-Hochberg-adjusted *P*-values, hypergeometric tests) vs. the proportion of EMT genes regulated by a microRNA. microRNAs which had significant EMT genes over-representation (Benjamini-Hochberg-adjusted *P* < 0.05) or regulate over half of EMT genes of are color-coded. **c** miR-508-3p expression is significantly lower in the mesenchymal subtype in TCGA dataset than the other three subtypes (*n* = 104 for mesenchymal, *n* = 124 for differentiated, *n* = 133 for proliferative, and *n* = 101 for immunoreactive). **d** Compared to non-mesenchymal subtypes, miR-508-3p expression is significantly lower in the mesenchymal subtype of ovarian cancer in TCGA (*n* = 104 for mesenchymal, *n* = 358 for non-mesenchymal). **e** miR-508-3p expression in ovarian cancer patients with relapse are significantly lower than those without relapse in Bagoli (*n* = 29 for non-relapse, *n* = 101 for relapse), OV133 (*n* = 39 for non-relapse, *n* = 94 for Relapse) and OV179 (*n* = 55 for non-relapse, *n* = 124 for relapse) datasets. **f** The expression levels of miR-508-3p in recurrent tumors (*n* = 8) are significantly lower than primary tumors (*n* = 8) and normal ovarian tissues (*n* = 4). **g** The expression levels of miR-508-3p are significantly higher in sensitive tumors (*n* = 69), compared to partial sensitive tumors (*n* = 26) and resistant tumors (*n* = 35). In all bar plots, *p*-values were based on Mann–Whitney-Wilcoxon test (*P < 0.05, **P < 0.01, ***P < 0.001). **h** Kaplan–Meier curves demonstrating OS and PFS of ovarian cancer patients in miR-508-3p low and high expression subgroups (stratified by the average expression level of miR-508-3p) in a merged cohort of three public datasets (Bagoli, OV133 and OV179). Using West China cohort (*n* = 131) for in-house validation, Kaplan–Meier curves confirmed significantly poorer overall survival in miR-508-3p low expression subgroup (also stratified by the average expression level of miR-508-3p) than tumors with high expression of miR-508-3p. *P*-values were based on log-rank tests

To elucidate regulatory mechanisms underlying the mesenchymal OvCa subtype, we inferred a microRNA regulatory network by integrative analysis of gene expression and microRNA expression profiles in the TCGA dataset (Fig. 1a). To identify putative master regulators for the mesenchymal subtype, master regulator analysis [14] was subsequently performed by testing overrepresentation of each microRNA’s regulon for EMT signature genes [15]. Using the strategy, we found miR-508-3p is the most statistically significant master regulator (Benjamini-Hochberg *P* = 0.019, a hypergeometric test) (Fig. 1a, b). Compared to non-mesenchymal subtypes, we observed significantly lower expression of miR-508-3p in the mesenchymal subtype in TCGA dataset (Fig. 1c, d).

### miR-508-3p is a strong indicator for clinical outcome of ovarian cancer patients

We further observed in three independent datasets, Bagnoli [16], OV133 [17] (GSE73582), and OV179 [17] (GSE73581), that miR-508-3p expression level was significantly lower in primary tumors of OvCa patients with relapse compared with those without (Fig. 1e). Interestingly, in recurrent OvCa tumors, miR-508-3p expression level was significantly lower than primary tumors and normal ovarian tissues (Fig. 1f). Furthermore, the expression level of miR-508-3p was significantly higher in chemosensitive OvCa than that in both partial chemosensitive OvCa and chemoresistant OvCa (Fig. 1g). Moreover, in a combined dataset of Bagnoli (*n* = 130), OV133 (*n* = 133) and OV179 (*n* = 179), miR-508-3p expression is prognostic of both overall survival (OS) and progression-free survival (PFS) (Fig. 1h and Supplementary Table S5). As our in-house validation, we examined the expression of miR-508-3p in the FFPE specimens of OvCa patients in West China cohort using qPCR analysis (Supplementary Table S4). We observed that lower expression of miR-508-3p was associated with significantly poorer OS and PFS (Fig. 1h). In both the three public datasets and West China cohort, the expression of miR-508-3p is predictive of survival independent of pathological stages and grades (Supplementary Fig. S2a, b and Supplementary Table S6).

### miR-508-3p inhibition induced EMT of non-mesenchymal subtype ovarian cancer cells

**Fig. 2:**
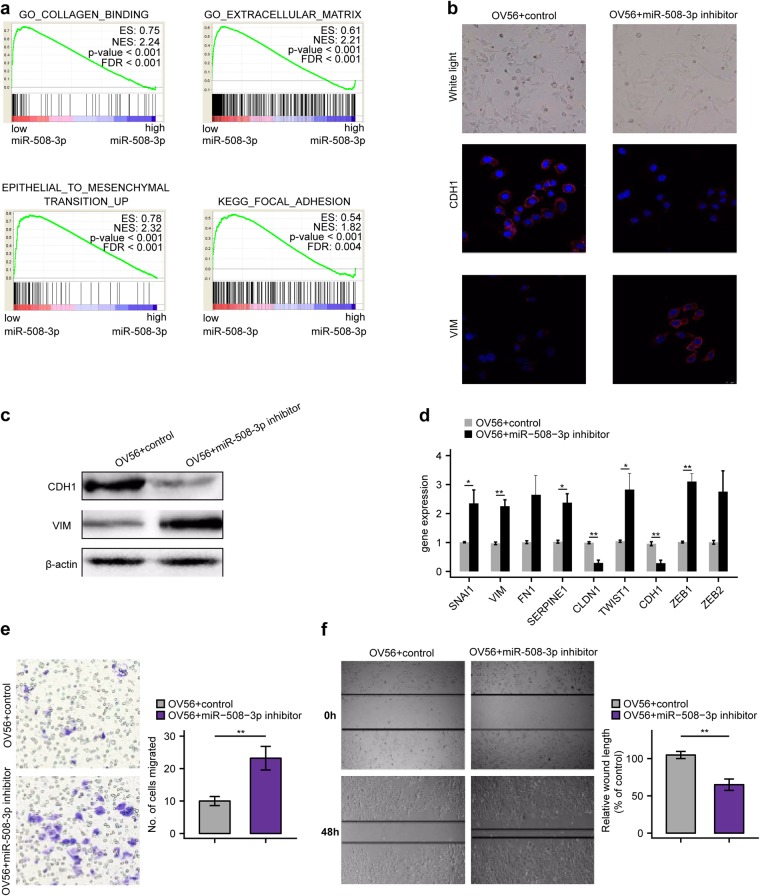
Inhibition of miR-508-3p in ovarian cancer cells is sufficient to induce mesenchymal phenotype. **a** Gene set enrichment analysis confirmed EMT-related programs are upregulated in miR-508-3p low expression group in TCGA. **b** Inverse phase microscopy (upper panel) and E-cadherin and vimentin staining (lower panels) of OV56 cells transfected with miR-508-3p inhibitor or control miRNA (miR-Ctrl) for 72 h. Cell nuclei were stained with DAPI. **c** The protein levels of E-cadherin and vimentin in OV56 cells transfected with miR-508-3p inhibitor or control miRNA (miR-Ctrl) for 72 h. β-actin was used as a control. **d** The mRNA levels of EMT genes between OV56 cells transfected with miR-508-3p inhibitor or control miRNA (miR-Ctrl) for 72 h. β-actin was used as a control. **e** Transwell chamber analysis of OV56 cells transfected with miR-508-3p inhibitor or control miRNA (miR-Ctrl) for 72 h. **f** Wound healing analysis of OV56 cells transfected with miR-508-3p inhibitor or control miRNA (miR-Ctrl) for 72 h. In all bar plots, *p*-values are based on two-tailed Student’s *t*-tests (*P < 0.05, **P < 0.01)

We next investigated whether miR-508-regulated transcriptome is enriched in mesenchymal-related molecular programs. Gene set enrichment analysis (GSEA) on the TCGA dataset revealed that genes comprising the signatures of collagen binding, extracellular matrix, EMT and focal adhesion, four programs widely accepted for their important role in mesenchymal feature, were highly enriched for OvCa with differential miR-508-3p expression levels (Fig. 2a and Supplementary Table S7). Next, we employed OV56 as the representative non-mesenchymal subtype OvCa cell line for functional study (the information for molecular subtyping of OvCa cell lines is shown in Supplementary Table S8) and transfected OV56 cells with miR-508-3p inhibitor and control(Supplementary Fig. S3a). We observed that the morphology of OV56 transfected with miR-508-3p inhibitor became spindle-like compared with control, accompanied with decreased expression level of epithelial marker E-cadherin and increased level of mesenchymal marker vimentin using immunofluorescent analysis (Fig. 2b). Consistently, western blotting analysis also revealed similar alterations on the protein level (Fig. 2c). In addition, the mRNA levels of mesenchymal markers including SNAI1, VIM, SERPINE1, TWIST1, and ZEB1 were significantly increased while epithelial markers including CLDN1 and CDH1 were significantly decreased in miR-508-3p inhibitor-transfected OV56 cells compared with control-transfected OV56 cells (Fig. 2c). Functionally, transfection of miR-508-3p inhibitor significantly enhanced migration ability of OV56 OvCa cells (Fig. 2e, f).

Next, we examined whether miR-508-3p could regulate the 10 genes (FBN1, SNAI2, CTSK, SEPT11, COL5A2, LUM, COL6A3, COL1A2, COL3A1, and SPARC) that classify the mesenchymal subtype. Using qPCR analysis, we demonstrated that miR-508-3p inhibition could induce upregulation of all the 10 genes in OV56 cells, although only the differences in 8 genes including FBN1, SNAI2, SEPT11, LUM, COL6A3, COL1A2, COL3A1, and SPARC showed statistical significance (Supplementary Fig. S3b). We subsequently performed RNA sequencing analysis of OVTOKO cells and OVISE cells transfected with either miR-508-3p inhibitor or control. We observed upregulation of EMT marker genes including SNAI1, fibronectin and SERPINE1 in OVTOKO cell line and SNAI1, FN1, SERPINE1, ZEB1 and ZEB2 in OVISE cell line (Supplementary Fig. S4a). GSEA coupled with Enrichment Map visualization [18] analysis was used to annotate and visualize differentially enriched biological pathways. We noticed enrichment of mesenchymal-related pathways including EMT, cell adhesion, cell migration, metastasis, and TGFB1 in miR-508-3p inhibitor-treated OvCa cell lines (Supplementary Fig. S4b and Supplementary Table S9). Representative pathways include extracellular matrix, TGFB1 signaling, EMT and cell migration programs in both OVTOKO and OVSIE cell lines (Supplementary Fig. S4c).

### Overexpression of miR-508-3p hampered EMT of ovarian cancer cells in vitro

**Fig. 3:**
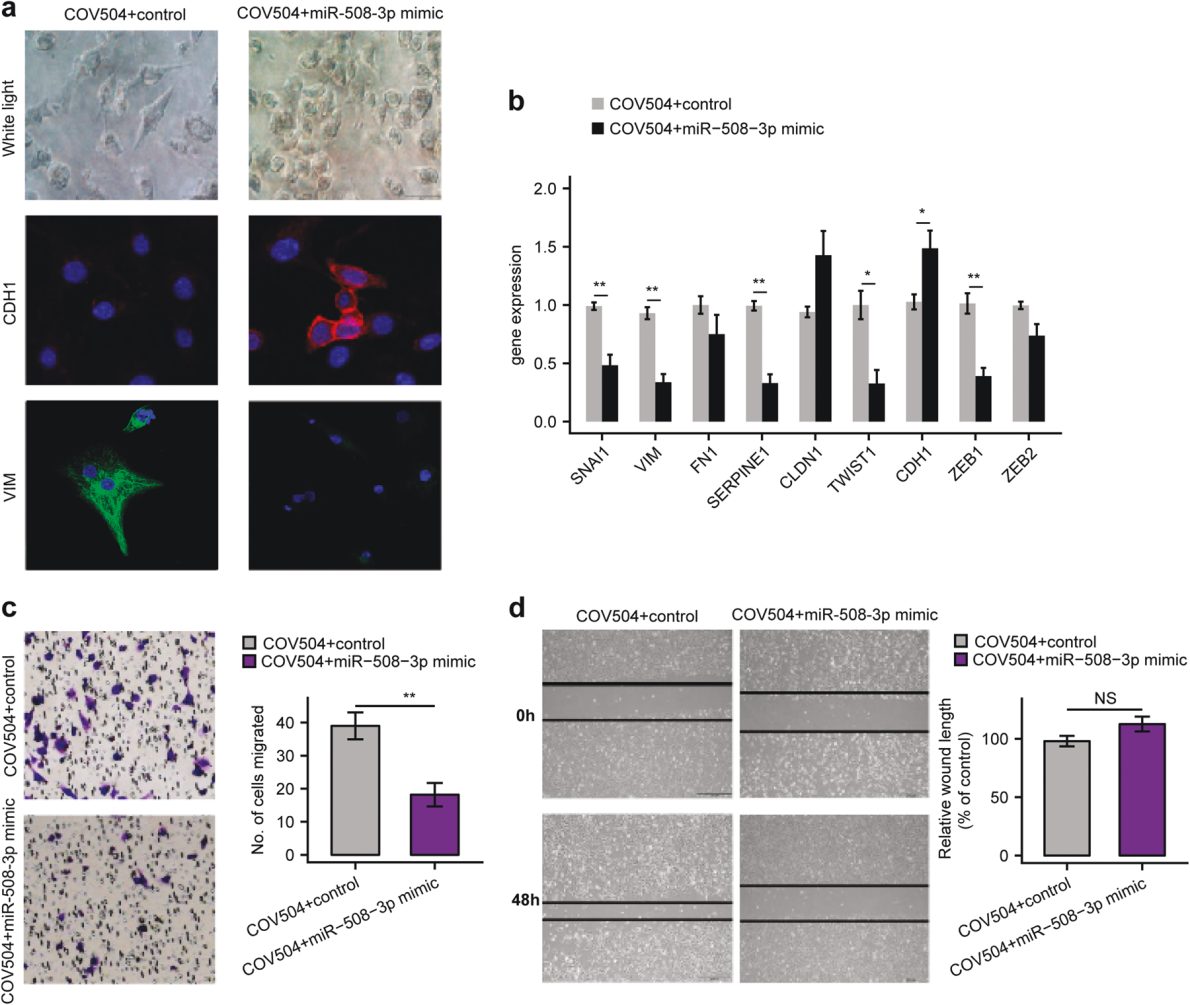
Enforced miR-508-3p expression reversed the mesenchymal identify of the mesenchymal subtype ovarian cancer. **a** Inverse phase microscopy (upper panel) and E-cadherin and vimentin staining (lower panels) of COV504 cells transfected with miR-508-3p mimic or control. Cell nuclei were stained with DAPI. **b** mRNA levels of key EMT genes in COV504 cells from the same transfection and treated the same way as described above. **c** Transwell chamber assay of the COV504 cells from the same transfection as described previously. **d** Wound healing analysis of COV504 cells transfected with miR-508-3p mimic or control for 72 h. In all bar plots, *P*-values are based on two-tailed Student’s *t*-tests (*P < 0.05, **P < 0.01)

We next asked whether enforced miR-508-3p expression in mesenchymal subtype OvCa cell lines could reverse its mesenchymal identity. We chose COV504 as the representative mesenchymal subtype OvCa cell line for functional validation. The results showed that the spindle-like morphology of COV504 transfected with miR-508-3p mimic turned into epithelial morphology, accompanied with increased expression of E-cadheirn and decreased expression of vimentin compared with control using immunofluorescence analysis (Fig. 3a). We further found that the mRNA levels of mesenchymal markers including SNAI1, VIM, SERPINE1, TWIST1, and ZEB1 were significantly downregulated while epithelial markers including E-cadheirn were significantly increased in miR-508-3p mimic-transfected COV504 cells compared with control (Fig. 3b). Functionally, Transwell chamber analysis and wound healing analysis also revealed that enforced expression of miR-508-3p mimic significantly hampered the migration ability of COV504 cells after enforced expression of COV504 compared with control (Fig. 3c, d).

It is well accepted that transforming growth factor-β (TGF-β) is a robust EMT inducer both under physiological and pathological circumstances [19]. We further investigated whether miR-508-3p could affect TGF-β-induced EMT in non-mesenchymal subtype OvCa cell lines. We treated OV56 and OVTOKO cells with TGF-β after transfecting miR-508-3p mimic or control and observed under white light that in control OV56 cells, TGF-β treatment induced elongated morphology of OV56 cell lines while miR-508-3p mimic transfection abolished this morphological alteration (Supplementary Fig. S5a, d). Moreover, after TGF-β treatment, OV56 cells transfected with control microRNA showed absence of membranous E-cadherin and remarkable induction of vimentin in the cytoplasm using immunofluorescence. By contrast, miR-508-3p mimic transfection attenuated the TGF-β-induced mesenchymal phenotype in OV56 cells, inducing an epithelial phenotype defined by E-cadherin localization at cell-cell junctions and reduced vimentin staining in the cytoplasm (Supplementary Fig. S5a). On the mRNA level, we noticed that TGF-β does significantly increase the mRNA levels of mesenchymal markers, including FN1, SERPINE1, and mesenchymal phenotype-specific transcription factors such as ZEB1 and ZEB2. Conversely, enhanced miR-508-3p expression in these cells reversed these mRNA changes (Supplementary Fig. S5b). Functionally, miR-508-3p treatment blocked TGF-β-induced OvCa migration using Transwell chamber analysis (Supplementary Fig. S5c). Similar results were also observed in OVTOKO cells (Supplementary Fig. S5d-f).

### LOX as a bona fide target of miR-508-3p in determining the mesenchymal identity of ovarian cancer

**Fig. 4:**
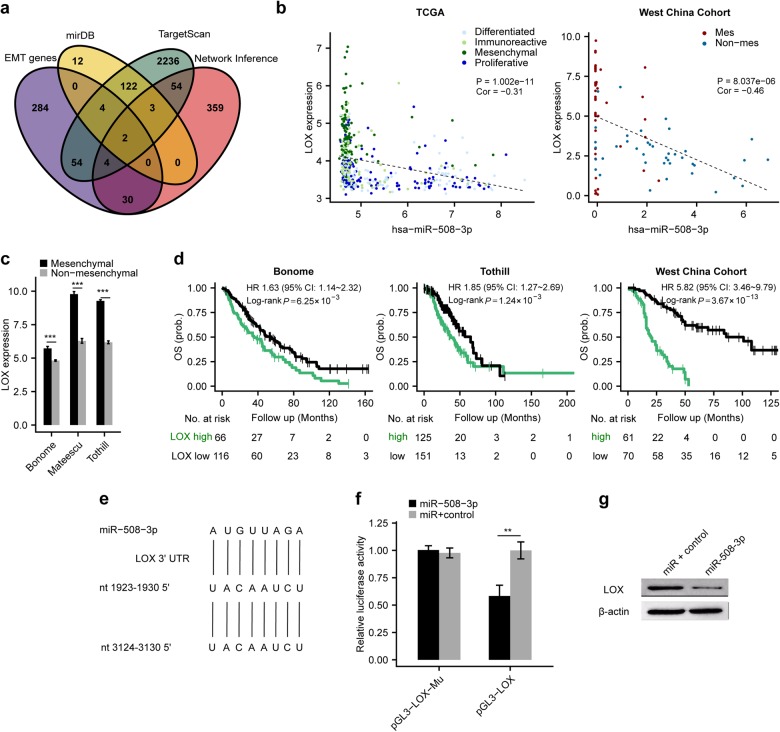
LOX is a downstream target of miR-508-3p in maintaining the mesenchymal identity of ovarian cancer. **a** Venn diagram illustrating miR-508-3p target prediction by taking the common genes between predicted target genes by miRDB and TargetScan databases, EMT signature genes and predicted target genes in the regulatory network. **b** Significant correlation between LOX expression and miR-508-3p expression in the TCGA dataset and West China cohort. r, Pearson correlation coefficient. **c** The expression of LOX is significantly higher in mesenchymal tumors than non-mesenchymal tumors in Bonome, Mateescu, and Tothill datasets. *P*-values are based on Mann–Whitney-Wilcoxon Test (***P < 0.001). **d** Kaplan–Meier curves showed significantly poorer overall survival in LOX high expression subgroup (stratified by the average expression level of LOX) than LOX low expression subgroup in Bonome, Tothill and West China cohorts, respectively. *P*-values are based on log-rank tests. **e** The two predicted binding sites of miR-508-3p were shown in the LOX 3′-UTR region. **f** The pGL3-LOX reporter gene has the full length of LOX 3′-UTR cloned into pGL3-control vector. The pGL3-LOX-Mu vector has the two miR-508-3p binding sites deleted and confirmed by sequencing OV56 cells were transfected with pGL3-LOX or pGL3-LOX-Mu, respectively, together with miR-508-3p mimic or negative control. *P*-values are based on two-tailed Student’s *t*-tests (**P < 0.01). **g** The expression level of LOX in OV56 cells transfected with miR-508-3p mimic or control

We further attempted to identify the potential target genes of miR-508-3p in OvCa. Using a strategy looking for the overlap between EMT signature genes, predicted target genes of miR-508-3p in the regulatory network and predicted microRNA targets by TargetScan [20] and mirDB [21] databases, we found two genes might be the direct targets of miR-508-3p, lysyl oxidase (LOX) and zinc finger E-box-binding homeobox 1 (ZEB1) (Fig. 4a). Using Pearson correlation analysis, we found that in TCGA dataset, the expression of miR-508-3p was negatively correlated with the expression of both LOX (Fig. 4b) and ZEB1 (Supplementary Fig. S6a). As our in-house validation, we also observed negative correlation between miR-508-3p and LOX expression in West China cohort (Supplementary Fig. S5b and Supplementary Table S4). Consistently, LOX expression in the tissue of mesenchymal OvCa subtype was significantly higher than non-mesenchymal tumors in Bonome dataset, Mateescu dataset and Tothill datasets (Fig. 4c). Furthermore, we observed that patients with higher expression of LOX displayed a significantly poorer OS in both Bonome and Tothill datasets as well as West China cohort (Fig. 4d). However, the expression level of ZEB1 is not associated with overall survival in either Bonome or Tothill dataset (Supplementary Fig. S6b).

It was predicted that three binding sites of miR-508-3p exist in the 3′-UTR of the LOX gene and we showed two of the binding sites in Fig. 4e. Subsequently, we conducted luciferase reporter assay to explore if miR-508-3p could directly target LOX. We cloned LOX 3′-UTR into the pGL3-ctrl vector and produced pGL3-LOX constructs. Cotransfection of pGL3-LOX and miR-508-3p mimic led to a 47.8% decrease in luciferase activity compared with control, implying that miR-508-3p directly targets LOX (Fig. 4f). To further validate that miR-508-3p specifically controls LOX expression via the predicted binding sites, we generated the control construct-pGL3-LOX-Mu, in which the miR-508-3p binding site sequences on LOX 3′-UTR were deleted. Subsequently, cotransfection of this construct with miR-508-3p mimic or miR-Ctrl into cells was conducted. It was observed that deletion of the miR-508-3p binding sites from the 3′-UTR of LOX attenuated the effects of miR-508-3p on luciferase activity (Fig. 4f). We further observed that transfection of miR-508-3p mimic in OV56 cells resulted in significant decrease of LOX protein expression (Fig. 4g).

To confirm that down-regulation of LOX is responsible for miR-508-3p-suppressed EMT and invasion, we further performed functional rescue experiments. We analyzed the migratory abilities of ovarian cancer cells through Transwell chamber analysis in control or LOX silenced OV56 cells transfected with miR-508-3p inhibitor or control or LOX-overexpressing COV504 cells transfected with miR-508-3p mimic. We noticed that silencing LOX could significantly reduce the migratory ability of miR-508-3p inhibitor-transfected OV56 cells compared with control(Supplementary Fig. S7a) while enforced LOX expression in miR-508-3p mimic-transfected COV504 cells significantly enhanced their migratory ability compared with control(Supplementary Fig. S7b). These observations demonstrated that LOX is a bona fide target of miR-508-3p in determining the mesenchymal identity of ovarian cancer.

### miR-508-3p inhibition triggered EMT program in vivo and in clinical specimens

**Fig. 5:**
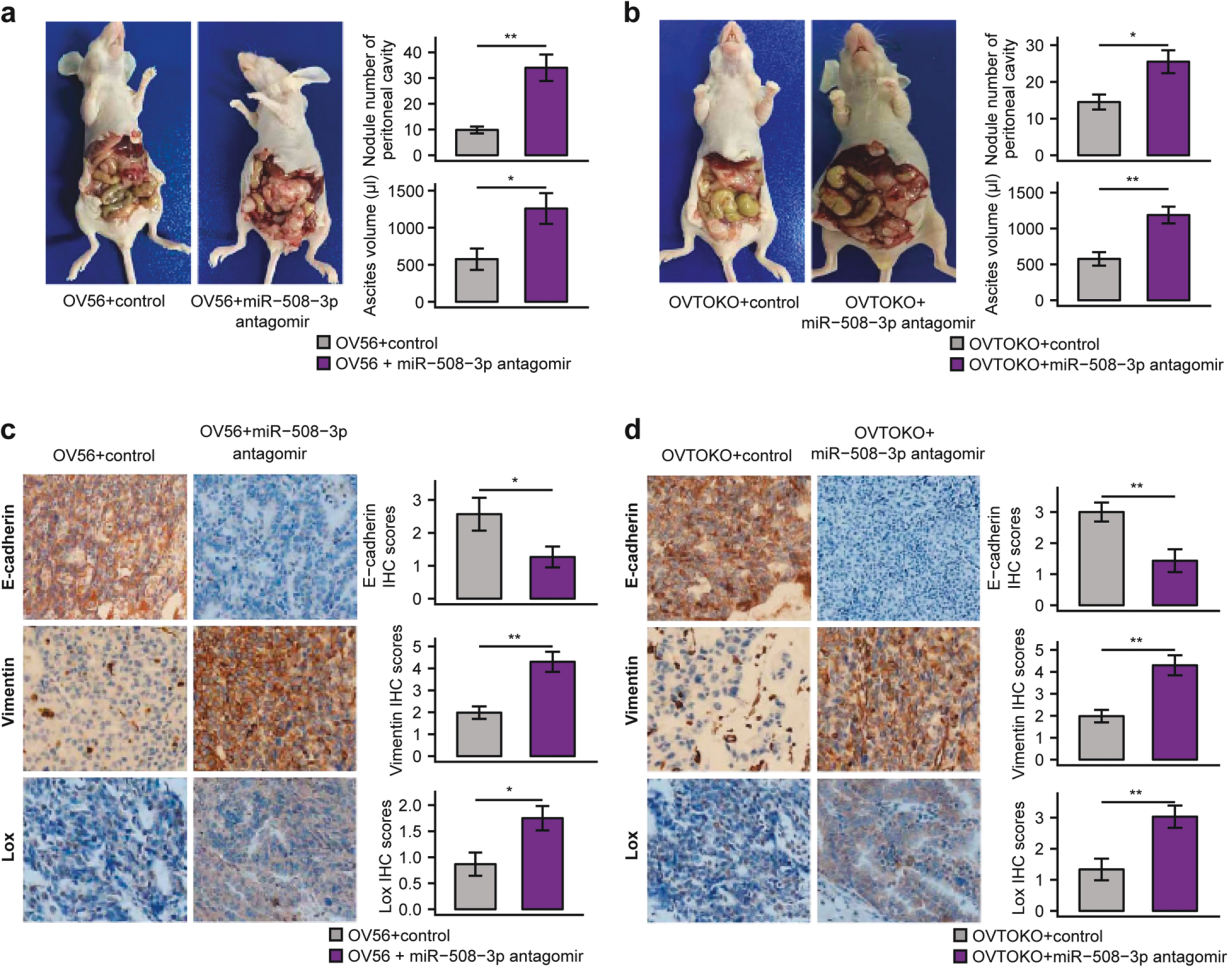
miR-508-3p inhibition promotes tumor progression in the peritoneal metastasis model of ovarian cancer. **a** Representative images of peritoneal metastasis and quantification of metastatic nodule number and ascites volume in control miRNA- and miR-508-3p antagomir-treated OV56 mouse model. **b** Representative images of peritoneal metastasis and quantification of metastatic nodule number and ascites volume in control miRNA- and miR-508-3p antagomir-treated OVTOKO mouse model. **c** OV56 tumor samples from control and miR-508-3p antagomir treated mice were stained for E-cadherin, vimentin and LOX by IHC. **d** OVTOKO tumor samples from control and miR-508-3p antagomir treated mice were stained for E-cadherin, vimentin and LOX by IHC. *P*-values are based on two-tailed Student’s *t*-tests (*P < 0.05, **P < 0.01, ***P < 0.001)

We further established an in vivo model using two non-mesenchymal subtype OvCa cell lines, OV56, and OVTOKO. For both models, delivery of miR-508-3p antagomir led to significant increase in the number of metastatic nodules and ascites volume compared with control (Fig. 5a, b). On the molecular level, compared with miR-Ctrl, miR-508-3p antagomir treatment significantly reduced expression of E-cadherin and induced expression of vimentin and LOX in vivo in both mouse models using immunostaining assays (Fig. 5c, d).

**Fig. 6:**
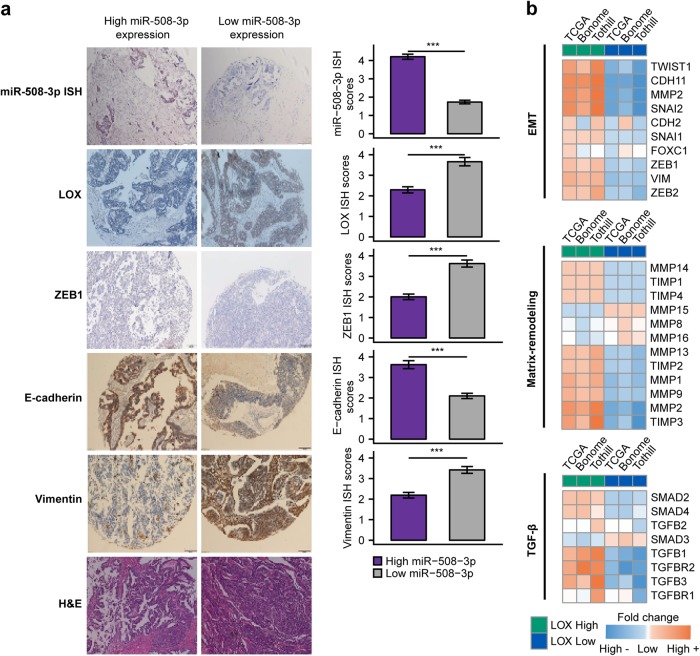
Correlation between miR-508-3p and E-cadherin, VIM, LOX and ZEB1 expression and in West China serous ovarian cancer cohorts. **a** Representative images of ISH staining for miR-508-3p and IHC staining for LOX, ZEB1, E-cadherin, and vimentin in miR-508-3p-low and -high expression cases. The miR-508-3p expression was stratified by using average of miR-508-3p qPCR expression as threshold. *P*-values are based on two-tailed Student’s *t*-tests (*P < 0.05, **P < 0.01, ***P < 0.001). **b** Heatmap showing the average log2 fold difference of the indicated genes (rows) in EMT-related programs (top: EMT, middle: matrix remodeling, bottom: TGF-β) between LOX-high and LOX-low ovarian cancer specimens of each respective dataset (columns)

We further analyzed the expression of miR-508-3p and EMT-related markers in tissue microarrays of OvCa patients from West China cohort. We found that the expression levels of LOX, ZEB1, and vimentin were significantly higher in the human OvCa tissues with lowly expressed miR-508-3p. By contrast, CDH1 expression was significantly lower in human OvCa tissues with low expression of miR-508-3p (Fig. 6a). Our further analysis of public datasets found that high LOX expression was significantly associated with EMT, matrix-remodeling, and TGF-β programs (Fig. 6b), indicating that LOX, as a bona fide target of miR-508-3p, could potentially determine the mesenchymal identity of OvCa.

### miR-508-3p loci methylation is a determinant of the mesenchymal subtype of ovarian cancer

We further examined the underlying mechanisms responsible for downregulation of miR-508-3p expression in the mesenchymal subtype of ovarian cancer. We were particularly interested in examining whether promoter methylation existed in the miR-508-3p loci as regulator of subtype-specific gene expression. In vitro, we observed that treatment of 5-aza-dC led to demethylation of the miR-508-3p loci in the mesenchymal cell line COV-504, resulting in upregulation of miR-508-3p expression (Supplementary Fig. S8a). Compared with the non-mesenchymal cell lines, miR-508-3p displayed significantly higher promoter methylation and lower expression in the mesenchymal cell line (Supplementary Fig. S8b). Interfering with acetylation, another component of epigenetic gene regulation program, by using histone deacetylase inhibitor Panobinostat, did not alter the expression of miR-508-3p in a meaningful and consistent way (data not shown). Altogether, we demonstrated that methylation of the miR-508-3p loci is a determinant of the mesenchymal subtype of ovarian cancer.

## Discussion

Ovarian cancer is a heterogeneous entity consisting of over 15 distinct tumor subtypes based upon histological appearance [4]. Whole genome or transcriptome profiling revealed various genomic and transcriptional variability correlated with distinct OvCa histological subtypes and provided the basis for the molecular subtyping to stratify different OvCa patients [22, 23]. The differences in their genetic instability were proved to derive from chromosomal mutations, copy number change or subsequent transcriptional heterogeneity. For instance, serous borderline tumors or low-grade serous ovarian carcinoma (LGSOC) are characterized by frequent KRAS or BRAF mutations while mucinous tumors carry KRAS mutations and endometrioid ovarian tumors harbor PTEN mutations [3]. However, currently very few reports have demonstrated the relations between the existing immunoreactive, differentiated, proliferative and mesenchymal subgroups and the different histological subtypes of OvCa. One study conducted by Tothill et al demonstrated that improved clustering of gene expression data identified six molecular subtypes of serous and endometrioid ovarian cancer [5]. Among them, two subtypes exhibited serous low malignant potential and low-grade endometrioid subtypes, respectively and the other four subtypes demonstrated higher grade and advanced stage cancers of serous and endometrioid histotypes. So far, the cancer cell types that are investigated by the approach of molecular subtyping include OvCa [24], liver cancer [25, 26], colorectal cancer [22, 23], and medulloblastoma [27]. However, the subtype-specific molecular portraits, especially the worst or best prognosis subtype-specific biomarkers still remain obscure for most of these cancer cell types. Our network-based strategy represents a major advancement compared with traditional transcriptome-based molecular subtyping in OvCa, which has led to inconsistent clinical associations from distinct datasets [28, 29].

miR-508-3p is located on Xq27.3, a fragile site of the human X chromosome [30]. Currently, the biological functions of miR-508-3p far from clear and its functions in cancer development and progression remain obscure. While miR-508-3p level demonstrated significant decreased expression in renal cell carcinoma (RCC) [31] and gastric cancer [30], overexpression of mir-508-3p was correlated with poor survival and increased aggressiveness in esophageal squamous cell carcinoma [32]. Existing literature has reported the differential expression of miR-508-3p in different OvCa tissues. For instance, Yu and his colleagues proved that four downregulated miRNAs including miR-508-3 in advanced-stage ovarian serous carcinoma compared with early stage [33]. However, in this study, miR-508-3p did not show prognosis relevance. In another study, Vang et al investigated the dysregulated miRNAs between primary serous carcinoma and their corresponding omental metastases [34]. They demonstrated that miR-508-3p was downregulated in metastases, but no functional analysis was performed. Chan et al identified a panel of prognostic tumor-suppressor miRNAs including miR-508-3p and investigated their regulation in the invasive behavior of ovarian cancer cells [35]. Although they demonstrated that overexpression of miR-508-3p reduced invasion property, they did not consider tumor heterogeneity and detect miRNAs expression difference in tumor samples. Another study conducted by Pan and his colleagues depicted that miR-509-3p can weaken cell migration, invasion, and aggregation ability by targeting YAP1 [36]. As miR-508-3p is also a member of the Xq27.3 miR cluster, they accessed miR-508-3p in vitro and found a weaker effect on migration. All these previous studies showed miR-508-3p expression is associated with invasiveness, but did not provide mechanistic insights into the regulatory role of this miRNA. Different from all the previous studies, our study initially undertook an unbiased, systems level search for the key regulators of OvCa based upon the molecular subtyping theory. Notably, our network-based multidimensional integration of miRNA-level and mRNA-level data for the first time unveiled that miR-508-3p constitutes a central role in the regulation of core genes specific for the identity of mesenchymal subtype of OvCa. Further functional studies characterized methylation-regulated miR-508-3p silencing is a key event that mediated the EMT process and aggressiveness of OvCa. Our study provides novel insights into the critical role of miR-508-3p as a mesenchymal identity determinant for OvCa patients.

EMT represents a state in which under certain circumstances, epithelial cells lose their cell–cell junctions, and gain a spindle-like, highly metastatic phenotype, enabling them to escape from the primary site and disseminate to distant organs [37, 38]. LOX is produced as 50 kDa proenzime, secreted, and then processed into a 32 kDa mature enzyme and a 18 kDa propeptide. Significant evidence has supported the function of the 32 kDa mature enzyme in mediating tumor progression via triggering matrix stiffening. These ECM alterations in cancer microenvironment could, in turn, direct cancer cell activities towards invasion, and migration [39, 40]. In this report, we demonstrated that LOX is a bona fide target of miR-508-3p, which could potently suppress the mesenchymal phenotype and TGF-β-induced EMT. Thus collectively, our study reveals that the miR-508-3p-based network is a determinant for the mesenchymal OvCa subtype and represents strong predictor of this subtype, which may constitute a new strategy for the management of the most aggressive subtype of OvCa patients.

## Materials and methods

### Patient series

In this study, we analyzed five independent miRNA datasets involving a total of 999 patients (Supplementary Table S1). In short, The Cancer Genome Atlas (TCGA) dataset includes 537 ovarian cancer patients [10]. The OV179 set comprises 179 ovarian cancer patients (GSE73581) [17]. The OV133 set (GSE73582) contains 133 ovarian cancer patients [17]. The Bagnoli set (GSE25204) comprises 130 ovarian cancer patients [16]. The Nam set (GSE83693) comprises 20 ovarian cancer patients [41]. Five independent mRNA datasets comprising a total of 1084 patients were used for this study. In short, the TCGA data set consists of 490 ovarian cancer patients [10]. The Bonome set (GSE26712) comprises 182 ovarian cancer patients [11]. The Tothill set (GSE9891) comprises 285 ovarian cancer patients [5]. The Mateescu set (GSE26193) comprises 107 ovarian cancer patients [42]. Finally, the West China cohort contains frozen and paraffin-embedded tissues from 131 ovarian cancer cases were collected from West China Second Hospital (Chengdu, China), which were approved by the Institutional Ethics Committee of Sichuan University. Informed consents from all patients were obtained before the study.

### Generation of the 10-gene classifier for subtype classification of validation datasets

To stratify ovarian cancer tumors into mesenchymal and non-mesenchymal subgroups, we conducted supervised analysis of the Bonome dataset (GSE26712) using subtyping information from Gottfried et al. [12]. The raw microarray data was first normalized and subsequently summarized using RMA [43], followed by *z*-score transformation. To build a classifier, we selected the most representative and predictive genes using a two-step filtering strategy. First, we used limma [44] (R package, version 3.32.2) to identify genes that are differentially expressed (log2 fold change > 1, FDR-adjusted *P* < 0.05) between ovarian cancer samples classified to the mesenchymal subtype and the others. Second, we calculated area under ROC curve using R package ROCR (version 1.0.7) to quantitate each gene’s discriminative power to separate the mesenchymal subtype from non-mesenchymal subtypes. Ten genes with AUC > 0.9 were used to train a classifier using PAM [13] method (Supplementary Table S2).

The 10-gene classifier was used to classify all public mRNA datasets, where a probability > 0.5 is indicative of subtype association. For all mRNA datasets, we used R package GEOquery [45] (version 1.0.7) to download RMA normalized gene expression data. For each dataset, the expression profiles were transformed from probe sets to genes, followed by z-normalization across all samples. The processed gene expression profiles were subsequently taken as input for classification using the 10-gene classifier established based on the Bonome dataset.

### Regulatory network inference

We performed regulatory network inference to study the relationships between microRNAs and potential targets by integrative analysis of gene expression profiles and microRNA expression profiles. To this end, we analyzed 462 patient samples with both microRNA and gene expression data in TCGA data set [10]. Thirteen microRNAs, downregulated (log2 fold change < −0.5, Benjamini-Hochberg-adjusted *P* < 0.0001) in tumors classified to the mesenchymal subtype compared to the others, were prioritized as potential regulators. 1672 genes differentially expressed between mesenchymal and non-mesenchymal tumors (absolute log2 fold change > 0.25, Benjamini-Hochberg-adjusted *P* < 0.05) were considered as potential targets of the seven microRNAs. The microRNA expression data and gene expression data were independently standard-normalized and subsequently integrated for network inference using RTN package [14]. Master regulator analysis was based on a hypergeometric test of overrepresentation of a microRNA’s predicted regulon, i.e., a set of target genes, for EMT signature genes [15]. Based on the analysis, hsa-miR-508-3p was identified as the most statistically significant master regulator of EMT (Benjamini-Hochberg-adjusted *P* = 0.019) in the mesenchymal ovarian cancer subtype.

### Gene set enrichment analysis

We performed gene set enrichment analysis using GSEA software [46] with 1000 permutations. For each data set, we took as input the phenotype by calculating the log2 fold change between gene expression profiles of mesenchymal samples and non-mesenchymal samples. We analyzed all gene sets included in the C2 and C5 databases downloaded from MSigDB (version 6). FDR-adjusted *P* < 0.05 or nominal (NOM) *P* < 0.05 were used to select statistically significant gene sets. Enrichment Map were done by HTSanalyzeR [18].

### Cell lines, cell culture, and cell transfection

The cell lines SKOV3, CAOV3, COV434, COV644, COV362, COV504 cells were cultured in DMEM supplemented with 10% fetal bovine serum (FBS), 2 mM l-glutamine, 1 mM sodium pyruvate and 100 U/ml penicillin-streptomycin mixture (both from Gibco-BRL, Grand Island, NY, USA). The cell lines A2780S, OVSAHO, OVTOKO, OV56, COLO-720E, OVISE, OV90, KURAMOCHI, OVCAR4, OVCAR3 were cultured in RPMI-1640 containing 10% FBS and 100 U/ml penicillin-streptomycin mixture (both from Gibco-BRL, Grand Island, NY, USA). We examned the cell lines received in 2014 for authenticity in 2016 by short tandem repeat (STR) genotyping.

miR-508-3p inhibitor, miR-508-3p antagomir and respective controls were obtained from RiboBio (Guangzhou, China). They were transfected into cells using Lipofectamine RNAiMAX (Invitrogen), respectively, based upon the manufacturer’s instructions. miR-508-3p mimic and control were also purchased from RiboBio (Guangzhou, China). Plasmid encoding full-length human LOX gene was purchased from Gene Copoeia (Guangzhou, China). They were transfected into cells using Lipofectamine 3000(Invitrogen), respectively, according to the manufacturer’s instructions.

### Quantitative reverse transcription PCR (qRT-PCR)

For qPCR of cellular mRNAs, RNA was isolated by a total RNA isolation kit (Norgen). For qPCR of mRNA derived from FFPE tissues, total RNA was extracted from four 10-μm FFPE tissue scrolls using the High Pure RNA paraffin kit (Roche Applied Science, Indianapolis, IN). The detailed procedures have been described previously [47]. Gene-specific primers as follows: miR-508-3p forward: 5′-CAAGCATGATTGTAGCCTTTTG-3′ and reverse: 5′-TATCGTTGTACTCCAGACCAAGAC-3′; snail forward: 5′-GAAAGGCCTTCAACTGCAAA-3′ and reverse: 5′-TGACATCTGAGTGGGTCTGG-3′; vimentin forward: 5′-GACAATGCGTCTCTGGCACGTCTT-3′ and reverse: 5′-TCCTCCGCCTCCTGCAGGTTCTT-3′; fibronectin forward: 5′-AAACTTGCATCTGGAGGCAAACCC-3′ and reverse: 5′-AGCTCTGATCAGCATGGACCACTT-3′; PAI-1 forward: 5′-GGCCATTACTACGACATCCTG-3′ and reverse: 5′-GGTCATGTTGCCTTTCCAGT-3′; claudin-1 forward: 5′-AACGCGGGGCTGCAGCTGTTG-3′ and reverse: 5′-GGATAGGGCCTTGGTGTTGGGT-3′; twist1 forward: 5′-GCAAGAAGTCGAGCGAAGAT-3′ and reverse: 5′-GCTCTGCAGCTCCTCGAA-3′; E-cadherin forward: 5′-GTCACTGACACCAACGATAATCCT-3′ and reverse: 5′-TTTCAGTGTGGTGATTACGACGTTA-3′; zeb1 forward: 5′-AGCAGTGAAAGAGAAGGGAATGC-3′ and reverse 5′-GGTCCTCTTCAGGTGCCTCAG-3′; zeb2 forward: 5′-CGCTTGACATCACTGAAGGA-3′ and reverse: 5′-CTTGCCACACTCTGTGCATT-3′; SNAI2 forward: 5′- CAGTGCAAAAACTGCTCCAA-3′ and reverse: 5′-GCTTCGGAGTGAAGAAATGC-3′; CTSK forward: 5′-CAGCAAAGGTGTGTATTATGATGAAAGC-3′ and reverse: 5′-ATGGGTGGAGAGAAGCAAAGTAGGAAGG-3′; SEPT11 forward: 5′-TTGGAGACCAGATAAATAAAGATGACA-3′ and reverse: 5′-CATGGTAGTTGAAGAGAGAACGTTTAA-3′; COL5A2 forwards: 5′-GCACGCTTGCCCATCATAGA-3′ and reverse: 5′-CCCAATTTCAACGCCGAATT-3′; LUM forward: 5′-CTTCAATCAGATAGCCAGACTGC-3′ and reverse: 5′-AGCCAGTTCGTTGTGAGATAAAC; COL6A3 forward: 5′-GAGCAGCTTGACAACATTGCC-3′ and reverse: 5′-GCCCAGAGCACTTGCAGG-3′; COL1A2 forward: 5′-CACCCAGAGTGGAGCAGTGG-3′ and reverse: 5′-TTCTTGGCTGGGATGTTTTCA-3′; COL3A1 forward: 5′-AATTTGGTGTGGACGTTGGC-3′ and reverse: 5′-TTGTCGGTCACTTGCACTG-3′; SPARC forward: 5′-AAACCGAAGAGGAGGTGGTG-3′ and reverse: 5′-GCAAAGAAGTGGCAGGAAGA-3′; GAPDH forward: 5′-ACCACAGTCCATGCCATCAC-3′ and reverse: 5′-TCCACCACCCTGTTGCTGTA-3′.

### Methylation PCR

Methylation PCR was performed as described previously [48]. Briefly, gDNA was isolated by the High Pure PCR Template Preparation Kit (Roche). Two micrograms of gDNA were bisulfite converted by the EpiTect Bisulfite Kit (Qiagen). Methylation levels were examined using the PyroMark PCR system (Qiagen).

### RNA sequencing

Whole transcriptome sequencing libraries were constructed as illustrated previously [49]. The libraries were sequenced on the Illumina HiSeq platform (Novogen, China). Reads were first trimmed to remove linker sequences and low-quality bases using Cutadapt [50] (version 1.2.1), and then mapped to the human reference genome (hg38), followed by calculation of gene counts using SATR [51] (version 2.5).

### miRNA in situ hybridization (ISH) and immunohistochemistry (IHC)

Paraffin-embedded ovarian cancer specimens from West China Second Hospital, Sichuan University were applied for ISH and IHC analysis. miRNA ISH assay was conducted as described previously [24]. In particular, the FFPE slides were hybridized with the double-DIG-labeled miRCURY LNATM detection probe, hsa-miR-508-3p(Exiqon), for 2 h at 55 °C (Ventana Discovery Ultra). IHC assay was performed as previously described [52]. The information of primary antibodies are as follows: E-cadherin (Abcam), Vimentin (Abcam), LOX (Abcam), and ZEB1 (Abcam). All slides were assessed by two independent pathologists in a double-blinded manner.

### Immunoflurescent analysis

Immunofluorescent analysis was performed as described previously [52]. The information of primary antibodies are as follows: E-cadherin (Abcam) and Vimentin (Abcam). Stained sections were viewed and photographed through a fluorescence microscope.

### Immunoblot

For immunoblotting, the whole cell lysates were prepared as described previously [47]. The primary antibodies used in immunoblotting analysis included: E-cadherin (Abcam), Vimentin (Abcam), and LOX(Abcam). The signals were quantified by QuantityOne software (Bio-Rad).

### An orthotopic intrabursal injection model of ovarian cancer in mice

In vivo assays were reviewed and approved by the Institutional Ethics Committee of Sichuan University. The female athymic BALB/c nude mice (6–8 weeks old, 18–20 g each) were used to evaluate the peritoneal metastatic potential of human ovarian cancer cell lines and they were randomly assigned to each group. The investigators were blinded to the group allocation during the experiment. An orthotopic model produced by intrabursal injection of ovarian cancer cell lines in mice was established as previously described [53].

### Transwell invasion assay and wound healing assay

Transwell chamber assay was applied for cell migration capacity assessment as described previously [54, 55]. As for wound healing assay, wounds were scratched in confluent cells by a pipette tip, and the cells were then rinsed with medium. Serum-free medium was next added, and culture plates were incubated at 37 °C. Wound healing process was observed at 0 and 48 h within the scrape line.

### Statistics

Kaplan–Meier curves were generated to show survival difference, and the significance was assessed by a log-rank test. Two-sided Student’s *t*-tests were used to assess differences in all experiments. Mann–Whitney–Wilcoxon tests were used for comparisons of different groups in all public cohorts. Univariate and multivariate Cox regression analyses were used to calculate hazard ratios (HRs) with 95% confidence intervals (CIs) to evaluate the prognostic significance of clinical factors and hsa-miR-508-3p expression level. Age, stage, and grade were potential confounding factors and were therefore included as covariates in the multivariate regression analysis. Statistical significance was denoted by *P < 0.05, **P < 0.01, ***P < 0.001, and a *P* value < 0.05 was considered to be significant. R (versions 3.4.0) was used for all statistical analyses described above.

## Electronic supplementary material

Supplementary information

Supplementary Fig.S1

Supplementary Fig.S2

Supplementary Fig.S3

Supplementary Fig.S4

Supplementary Fig.S5

Supplementary Fig.S6

Supplementary Fig.S7

Supplementary Fig.S8

Supplementary Table S1

Supplementary Table S2

Supplementary Table S3

Supplementary Table S4

Supplementary Table S5

Supplementary Table S6

Supplementary Table S7

Supplementary Table S8

Supplementary Table S9

## Data Availability

All high-throughput sequencing data has been deposited in GEO database (accession number: GSE108863).

## References

[1] Matulonis UA, Sood AK, Fallowfield L, Howitt BE, Sehouli J, Karlan BY (2016). Ovarian cancer. Nat Rev Dis Prim.

[2] Bowtell DD, Böhm S, Ahmed AA, Aspuria PJ, Bast RC, Beral V (2015). Rethinking ovarian cancer II: reducing mortality from high-grade serous ovarian cancer. Nat Rev Cancer.

[3] Karnezis AN, Cho KR, Gilks CB, Pearce CL, Huntsman DG (2017). The disparate origins of ovarian cancers: pathogenesis and prevention strategies. Nat Rev Cancer.

[4] Kossaï Myriam, Leary Alexandra, Scoazec Jean-Yves, Genestie Catherine (2017). Ovarian Cancer: A Heterogeneous Disease. Pathobiology.

[5] Tothill RW, Tinker AV, George J, Brown R, Fox SB, Lade S (2008). Novel molecular subtypes of serous and endometrioid ovarian cancer linked to clinical outcome. Clin Cancer Res.

[6] Rupaimoole R, Slack FJ (2017). MicroRNA therapeutics: towards a new era for the management of cancer and other diseases. Nat Rev Drug Discov.

[7] Nieto MA, Huang RYJ, Jackson RA, Thiery JP (2016). EMT: 2016. Cell.

[8] Lambert AW, Pattabiraman DR, Weinberg RA (2017). Emerging biological principles of metastasis. Cell.

[9] Bilyk O, Coatham M, Jewer M, Postovit LM (2017). Epithelial-to-mesenchymal transition in the female reproductive tract: from normal functioning to disease pathology. Front Oncol.

[10] Cancer Genome Atlas Research Network. (2011). Integrated genomic analyses of ovarian carcinoma. Nature.

[11] Bonome T, Levine DA, Shih J, Randonovich M, Pise-Masison CA, Bogomolniy F (2008). A gene signature predicting for survival in suboptimally debulked patients with ovarian cancer. Cancer Res.

[12] Konecny GE, Wang C, Hamidi H, Winterhoff B, Kalli KR, Dering J et al. Prognostic and therapeutic relevance of molecular subtypes in high-grade serous ovarian cancer. J Natl Cancer Inst. 2014; 10.1093/jnci/dju249. 10.1093/jnci/dju249 PMC4271115 25269487

[13] Tibshirani R, Hastie T, Narasimhan B, Chu G (2002). Diagnosis of multiple cancer types by shrunken centroids of gene expression. Proc Natl Acad Sci USA.

[14] Fletcher MNC, Castro MAA, Wang X, de Santiago I, O’Reilly M, Chin SF (2013). Master regulators of FGFR2 signalling and breast cancer risk. Nat Commun.

[15] Zhao M, Kong L, Liu Y, Qu H (2015). dbEMT: an epithelial-mesenchymal transition associated gene resource. Sci Rep.

[16] Bagnoli M, De Cecco L, Granata A, Nicoletti R, Marchesi E, Alberti P (2011). Identification of a chrXq27.3 microRNA cluster associated with early relapse in advanced stage ovarian cancer patients. Oncotarget.

[17] Bagnoli M, Canevari S, Califano D, Losito S, Maio MD, Raspagliesi F (2016). Development and validation of a microRNA-based signature (MiROvaR) to predict early relapse or progression of epithelial ovarian cancer: a cohort study. Lancet Oncol.

[18] Wang X, Terfve C, Rose JC, Markowetz F (2011). HTSanalyzeR: an R/Bioconductor package for integrated network analysis of high-throughput screens. Bioinformatics.

[19] Jiang J, Wang K, Chen Y, Chen H, Nice EC, Huang C (2017). Redox regulation in tumor cell epithelial-mesenchymal transition: molecular basis and therapeutic strategy. Signal Transduct Target Ther.

[20] Friedman RC, Farh KKH, Burge CB, Bartel DP (2009). Most mammalian mRNAs are conserved targets of microRNAs. Genome Res.

[21] Wong N, Wang X (2015). miRDB: an online resource for microRNA target prediction and functional annotations. Nucleic Acids Res.

[22] De Sousa E, Melo F, Wang X, Jansen M, Fessler E, Trinh A (2013). Poor-prognosis colon cancer is defined by a molecularly distinct subtype and develops from serrated precursor lesions. Nat Med.

[23] Guinney J, Dienstmann R, Wang X, de Reyniès A, Schlicker A, Soneson C (2015). The consensus molecular subtypes of colorectal cancer. Nat Med.

[24] Yang D, Sun Y, Hu L, Zheng H, Ji P, Pecot CV (2013). Integrated analyses identify a master microRNA regulatory network for the mesenchymal subtype in serous ovarian cancer. Cancer Cell.

[25] Cairo S, Armengol C, De Reyniès A, Wei Y, Thomas E, Renard CA (2008). Hepatic stem-like phenotype and interplay of Wnt/beta-catenin and Myc signaling in aggressive childhood liver cancer. Cancer Cell.

[26] Chaisaingmongkol J, Budhu A, Dang H, Rabibhadana S, Pupacdi B, Kwon SM (2017). Common molecular subtypes among Asian hepatocellular carcinoma and cholangiocarcinoma. Cancer Cell.

[27] Northcott PA, Buchhalter I, Morrissy AS, Hovestadt V, Weischenfeldt J, Ehrenberger T (2017). The whole-genome landscape of medulloblastoma subtypes. Nature.

[28] Tan TZ, Miow QH, Huang RYJ, Wong MK, Ye J, Lau JA (2013). Functional genomics identifies five distinct molecular subtypes with clinical relevance and pathways for growth control in epithelial ovarian cancer. EMBO Mol Med.

[29] Way GP, Rudd J, Wang C, Hamidi H, Fridley BL, Konecny GE (2016). Comprehensive cross-population analysis of high-grade serous ovarian cancer supports no more than three subtypes. G3.

[30] Huang T, Kang W, Zhang B, Wu F, Dong Y, Tong JHM (2016). miR-508-3p concordantly silences NFKB1 and RELA to inactivate canonical NF-κB signaling in gastric carcinogenesis. Mol Cancer.

[31] Zhai Q, Zhou L, Zhao C, Wan J, Yu Z, Guo X (2012). Identification of miR-508-3p and miR-509-3p that are associated with cell invasion and migration and involved in the apoptosis of renal cell carcinoma. Biochem Biophys Res Commun.

[32] Lin C, Liu A, Zhu J, Zhang X, Wu G, Ren P (2014). miR-508 sustains phosphoinositide signalling and promotes aggressive phenotype of oesophageal squamous cell carcinoma. Nat Commun.

[33] Yu X, Zhang X, Bi T, Ding Y, Zhao J, Wang C (2013). MiRNA expression signature for potentially predicting the prognosis of ovarian serous carcinoma. Tumour Biol.

[34] Vang S, Wu HT, Fischer A, Miller DH, MacLaughlan S, Douglass E (2013). Identification of ovarian cancer metastatic miRNAs. PLoS ONE.

[35] Chan Clara K., Pan Yinghong, Nyberg Kendra, Marra Marco A., Lim Emilia L., Jones Steven J. M., Maar Dianna, Gibb Ewan A., Gunaratne Preethi H., Robertson A. Gordon, Rowat Amy C. (2016). Tumour-suppressor microRNAs regulate ovarian cancer cell physical properties and invasive behaviour. Open Biology.

[36] Pan Y, Robertson G, Pedersen L, Lim E, Hernandez-Herrera A, Rowat AC (2017). Correction: miR-509-3p is clinically significant and strongly attenuates cellular migration and multi-cellular spheroids in ovarian cancer. Oncotarget.

[37] Ceppi P, Peter ME (2014). MicroRNAs regulate both epithelial-to-mesenchymal transition and cancer stem cells. Oncogene.

[38] Floor S, van Staveren WCG, Larsimont D, Dumont JE, Maenhaut C (2011). Cancer cells in epithelial-to-mesenchymal transition and tumor-propagating-cancer stem cells: distinct, overlapping or same populations. Oncogene.

[39] Liu N, Cox TR, Cui W, Adell G, Holmlund B, Ping J (2017). Nuclear expression of lysyl oxidase enzyme is an independent prognostic factor in rectal cancer patients. Oncotarget.

[40] Di Stefano V, Torsello B, Bianchi C, Cifola I, Mangano E, Bovo G (2016). Major action of endogenous lysyl oxidase in clear cell renal cell carcinoma progression and collagen stiffness revealed by primary cell cultures. Am J Pathol.

[41] Nam EJ, Kim S, Lee TS, Kim HJ, Lee JY, Kim SW (2016). Primary and recurrent ovarian high-grade serous carcinomas display similar microRNA expression patterns relative to those of normal ovarian tissue. Oncotarget.

[42] Mateescu B, Batista L, Cardon M, Gruosso T, de Feraudy Y, Mariani O (2011). miR-141 and miR-200a act on ovarian tumorigenesis by controlling oxidative stress response. Nat Med.

[43] Bolstad BM, Irizarry RA, Astrand M, Speed TP (2003). A comparison of normalization methods for high density oligonucleotide array data based on variance and bias. Bioinformatics.

[44] Ritchie ME, Phipson B, Wu D, Hu Y, Law CW, Shi W (2015). limma powers differential expression analyses for RNA-sequencing and microarray studies. Nucleic Acids Res.

[45] Davis S, Meltzer PS (2007). GEOquery: a bridge between the gene expression omnibus (GEO) and bioConductor. Bioinformatics.

[46] Subramanian A, Tamayo P, Mootha VK, Mukherjee S, Ebert BL, Gillette MA (2005). Gene set enrichment analysis: a knowledge-based approach for interpreting genome-wide expression profiles. Proc Natl Acad Sci USA.

[47] Zhao L, Ji G, Le X, Wang C, Xu L, Feng M (2017). Long noncoding RNA LINC00092 acts in cancer-associated fibroblasts to drive glycolysis and progression of ovarian cancer. Cancer Res.

[48] Zhou S, Li Y, Huang F, Zhang B, Yi T, Li Z (2012). Live-attenuated measles virus vaccine confers cell contact loss and apoptosis of ovarian cancer cells via ROS-induced silencing of E-cadherin by methylation. Cancer Lett.

[49] Yu Y, Chen Y, Kim B, Wang H, Zhao C, He X (2013). Olig2 targets chromatin remodelers to enhancers to initiate oligodendrocyte differentiation. Cell.

[50] Martin M (2011). Cutadapt removes adapter sequences from high-throughput sequencing reads. EMBnet J.

[51] Dobin A, Davis CA, Schlesinger F, Drenkow J, Zaleski C, Jha S (2013). STAR: ultrafast universal RNA-seq aligner. Bioinformatics.

[52] Zhou S, Yi T, Liu R, Bian C, Qi X, He X (2012). Proteomics identification of annexin A2 as a key mediator in the metastasis and proangiogenesis of endometrial cells in human adenomyosis. Mol Cell Proteom.

[53] Zhao L, Ji G, Le X, Luo Z, Wang C, Feng M (2017). An integrated analysis identifies STAT4 as a key regulator of ovarian cancer metastasis. Oncogene.

[54] Zhao L, Zhou S, Zou L, Zhao X (2013). The expression and functionality of stromal caveolin 1 in human adenomyosis. Hum Reprod.

[55] Zhao L, Wang W, Huang S, Yang Z, Xu L, Yang Q (2018). The RNA binding protein SORBS2 suppresses metastatic colonization of ovarian cancer by stabilizing tumor-suppressive immunomodulatory transcripts. Genome Biol.

