# Supplementary material for: Integrative network biology analysis identifies miR-508-3p as the determinant for the mesenchymal identity and a strong prognostic biomarker of ovarian cancer
Source: Oncogene. 2018 Nov 26;38(13):2305–19. doi: 10.1038/s41388-018-0577-5 (PMC6755993; doi:10.1038/s41388-018-0577-5)
Supplement: Supplementary file 1 — Supplementary information [file 41388_2018_577_MOESM1_ESM.docx]

**Supplementary information**

**Supplementary Fig. S1. Clinical ovarian cancer patients could be stratified into mesenchymal and non-mesenchymal subtypes with distinct clinical outcome.**

**a** Bonome cohort (*n* = 182) was used to build a 10-gene classifier to distinguish the mesenchymal subtype from the others. The top bar indicates the subtypes. In the heatmap, rows indicate genes from the classifier and columns represent patients. Kaplan-Meier curves showed four molecular subtypes (middle) and two subtypes (right) of Bonome cohort defined by Konecny *et al*, respectively. The mesenchymal subtype was significantly associated with poor survival. **b** Tothill cohort (*n* = 285) can be stratified into mesenchymal and non-mesenchymal subtypes (left) by the 10-gene classifier. Kaplan-Meier curves showed mesenchymal subtype was significantly associated with poor survival (right). **c** Mateescu cohort (*n* = 107) can also be stratified into mesenchymal and non-mesenchymal subtypes (left) by the 10-gene classifier. Kaplan-Meier curves showed mesenchymal subtype was significantly associated with poor survival (right). *P*-values are based on log-rank tests.

**Supplementary Fig. S2. The association of miR-508-3p expression with clinical survival of ovarian cancer patients.**

**a** Kaplan-Meier curves showed overall survival and progression-free survival of patients in miR-508-3p low and high expression subgroups (stratified by the average expression level of miR-508-3p) in OV133 (*n*=133), OV179 (*n*=179) and Bagoli (*n*=130) datasets, respectively. **b** Kaplan-Meier curves showed overall survival of patients in miR-508-3p low and high expression subgroups (stratified by the average expression level of miR-508-3p) in West China cohort (*n*=131) with different FIGO stages (left two panels) and pathological grades (right panel). *P*-values are based on log-rank tests.

**Supplementary Fig. S3. a** The mRNA levels of miR-508-3p in OV56 cells after transfection of control and miR-508-3p inhibitor. **b** The change of mRNA levels of the 10 genes that determine the mesenchymal identity(FBN1, SNAI2, CTSK, SEPT11, COL5A2, LUM, COL6A3, COL1A2, COL3A1 and SPARC) in OV56 cells after miR-508-3p inhibition. In all bar plots, *p*-values are based on two-tailed Student’s *t*-tests (**P* < 0.05, ***P* < 0.01, ****P* < 0.001).

**Supplementary Fig. S4. miR-508-3p inhibition induces EMT program in non-mesenchymal subtype ovarian cancer cells.**

**a** The mRNA levels of EMT markers dysregulated in OVTOKO cells (*n*=2, left panel) and OVISE cells (*n*=2, right panel) after transfection of miR-508-3p inhibitor. In all bar plots, *p*-values are based on two-tailed Student’s *t*-tests (**P* < 0.05, ***P* < 0.01, ****P* < 0.001). **b** Cellular programs enriched by GSEA in non-mesenchymal subtype ovarian cancer cells (OVTOKO and OVISE) transfected with miR-508-3p inhibitor represented using Enrichment Map. Node size represents the number of genes overlapped. **c** Representative GSEA enrichment plots. *P*-values are calculated by GSEA software.

**Supplementary Fig. S5. Overexpression of miR-508-3p alone in ovarian cancer cells is sufficient to block TGFβ-induced EMT.**

**a** Inverse phase microscopy (left panel) and E-cadherin and vimentin staining (right panels) of OV56 cells transfected with miR-508-3p or control miRNA (miR-Ctrl). At 24 hr post-transfection, the cells were cultured in serum-free medium containing TGF-β1 for 48 hr. Cell nuclei were stained with DAPI. **b** mRNA levels of key EMT-related genes in OV56 cells from the same transfection and treated the same way as described above. **c** Transwell chamber assay of the OV56 cells from the same transfection as described previously. At 24 hr post-transfection the cells were cultured in serum-free medium with TGF-β1 for 24 hr and then were seeded into triplicate invasion chambers in serum-free medium containing TGFb1. The cells were allowed to invade toward serum for 22 hr. **d** Inverse phase microscopy (left panel) and E-cadherin and vimentin staining (right panels) of OVTOKO cells transfected with miR-508-3p or control miRNA (miR-Ctrl). At 24 hr post-transfection, the cells were cultured in serum-free medium containing TGF-β1 for 48 hr. Cell nuclei were stained with DAPI. **e** mRNA levels of key EMT-related genes in OVTOKO cells from the same transfection and treated the same way as described above. **f** Transwell chamber assay of the OVTOKO cells from the same transfection as described previously. At 24 hr post-transfection the cells were cultured in serum-free medium with TGF-β1 for 24 hr and then were seeded into triplicate invasion chambers in serum-free medium containing TGFb1. The cells were allowed to invade toward serum for 22 hr. In all bar plots, *p*-values are based on two-tailed Student’s *t*-tests (**P* < 0.05, ***P* < 0.01, ****P* < 0.001).

**Supplementary Fig. S6. ZEB1 expression is inversely correlated with miR-508-3p, but not associated with overall survival.**

**a** Significant correlation between ZEB1 expression and miR-508-3p expression in TCGA dataset. r, Pearson correlation coefficient. **b** Kaplan-Meier curves showed no difference in overall survival between ZEB1 low and high expression subgroups (stratified by the average expression level of ZEB1) in TCGA dataset. *P*-values are based on log-rank tests.

**Supplementary Fig. S7. a** Transwell chamber analysis of control or LOX silenced OV56 cells transfected with miR-508-3p inhibitor. **b** Transwell chamber analysis of control or LOX-overexpressing COV504 cells transfected with miR-508-3p mimic. In all bar plots, *p*-values are based on two-tailed Student’s *t*-tests (**P* < 0.05, ***P* < 0.01, ****P* < 0.001).

**Supplementary Fig. S8. Methylation of the miR-508-3p loci is a determinant of the mesenchymal subtype of ovarian cancer**

**a** 5-aza treatment of the COV504 cell lines reveals that a reduction in methylation results in re-expression of miR-508-3p. **b** We measured the expression of miR-503-3p and the methylation of miR-508-3p loci between the non-mesenchymal subtype ovarian cancer cell lines and mesenchymal subtype ovarian cancer cell line. In all bar plots, *p*-values are based on two-tailed Student’s *t*-tests (**P* < 0.05, ***P* < 0.01, ****P* < 0.001).
