# Supplementary figures and images for: Integrative network biology analysis identifies miR-508-3p as the determinant for the mesenchymal identity and a strong prognostic biomarker of ovarian cancer
Source: Oncogene. 2018 Nov 26;38(13):2305–19. doi: 10.1038/s41388-018-0577-5 (PMC6755993; doi:10.1038/s41388-018-0577-5)

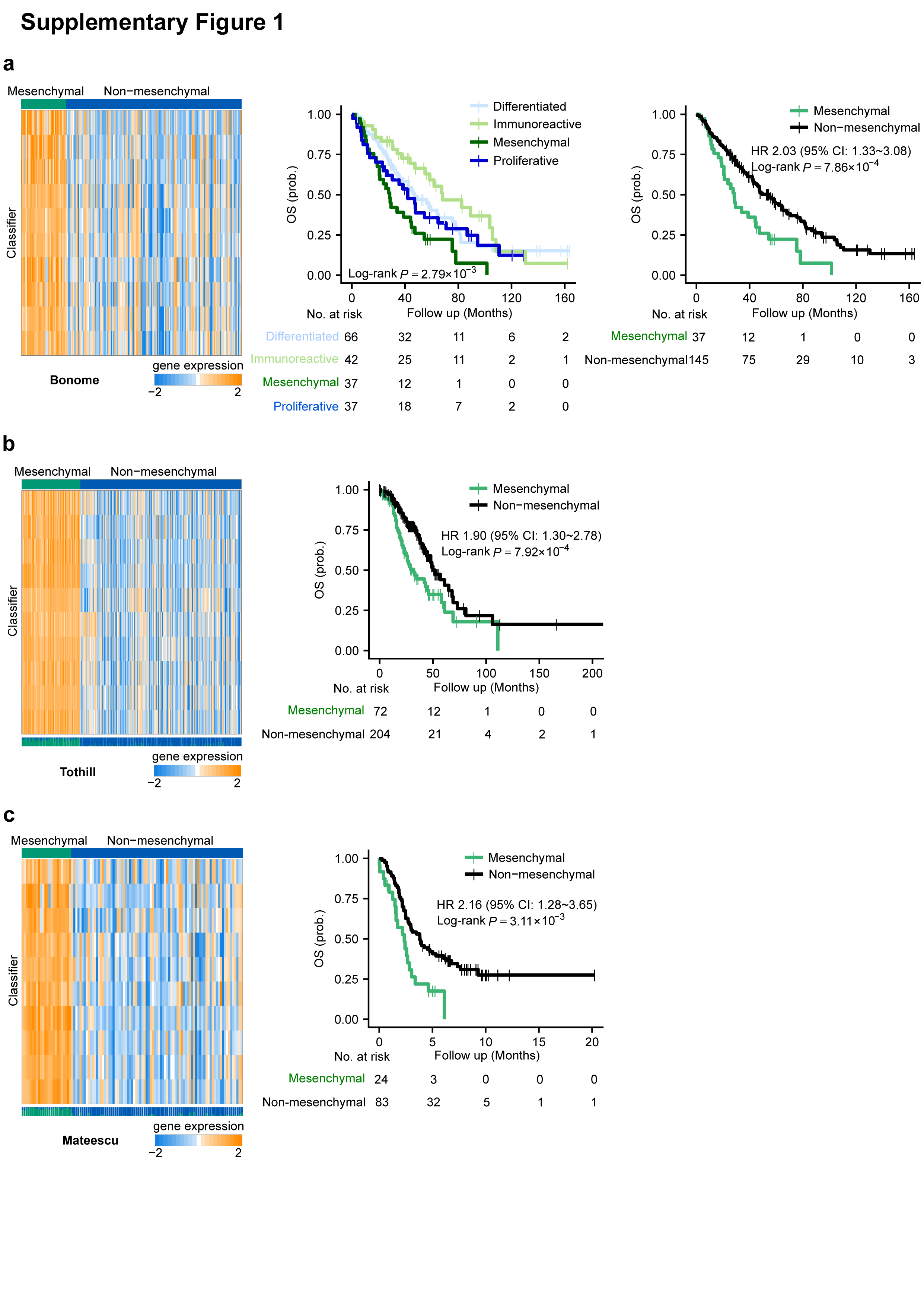

Supplement: Supplementary file 2 — Supplementary Fig.S1 [file 41388_2018_577_MOESM2_ESM.tif]

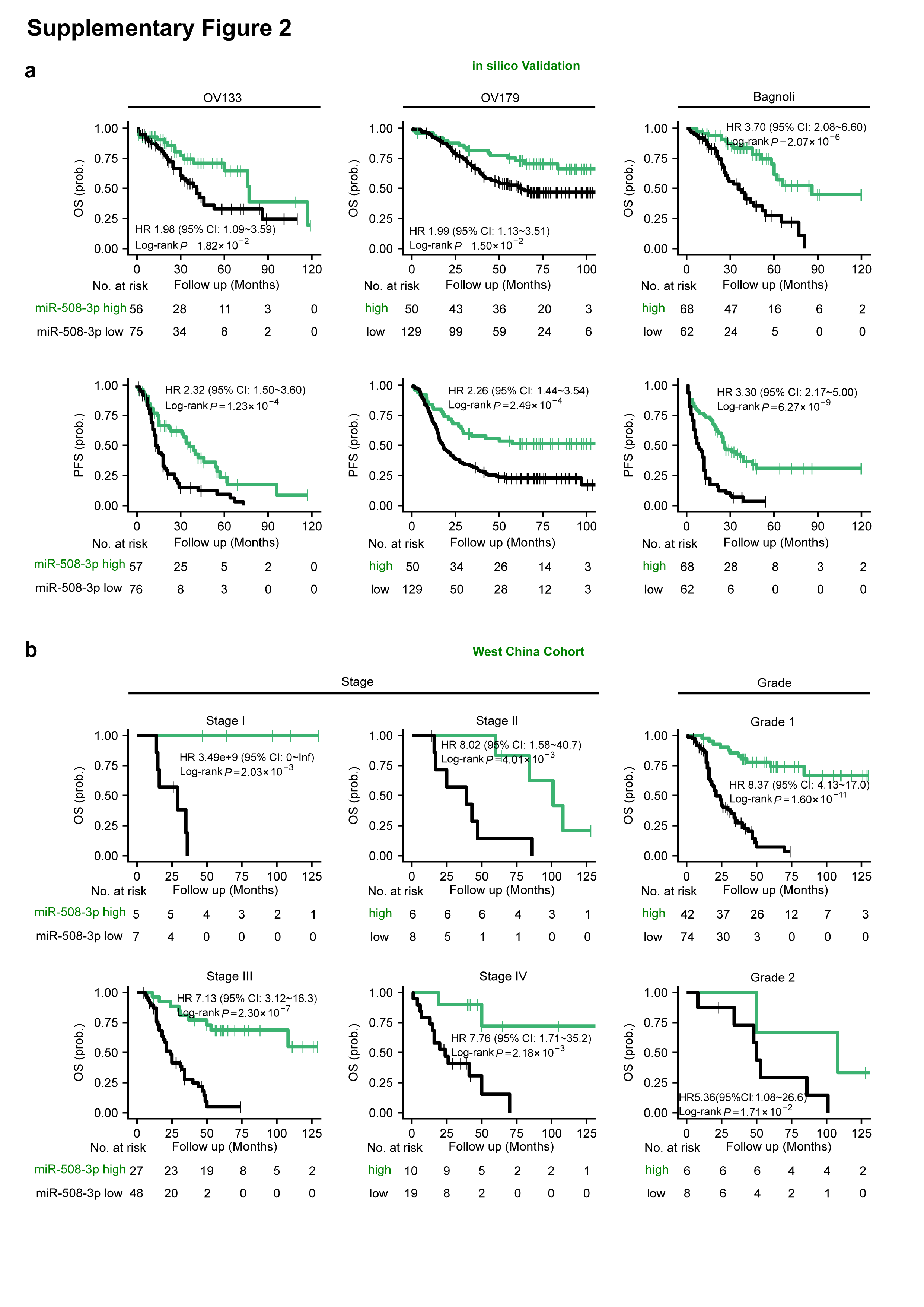

Supplement: Supplementary file 3 — Supplementary Fig.S2 [file 41388_2018_577_MOESM3_ESM.tif]

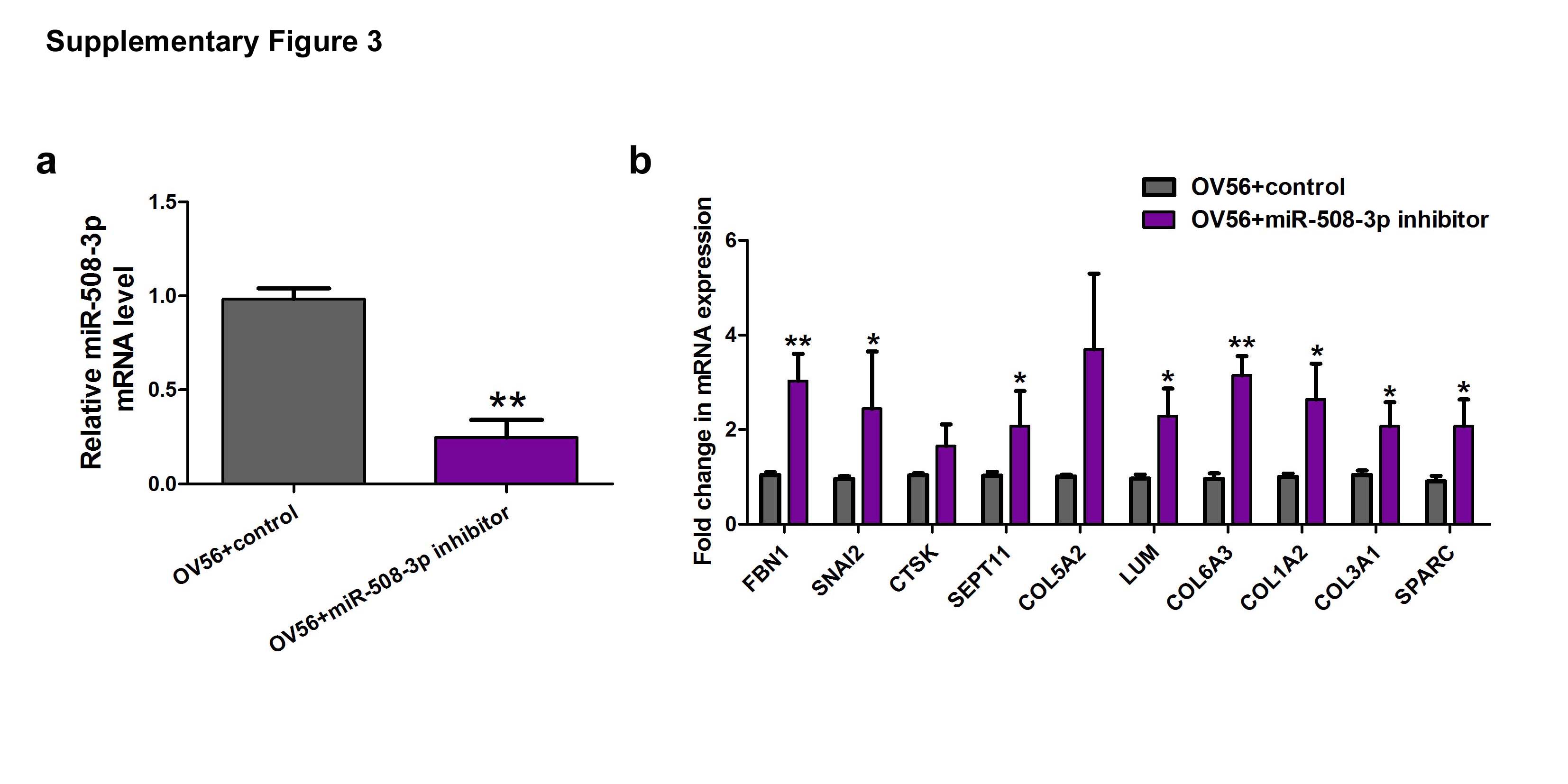

Supplement: Supplementary file 4 — Supplementary Fig.S3 [file 41388_2018_577_MOESM4_ESM.jpg]

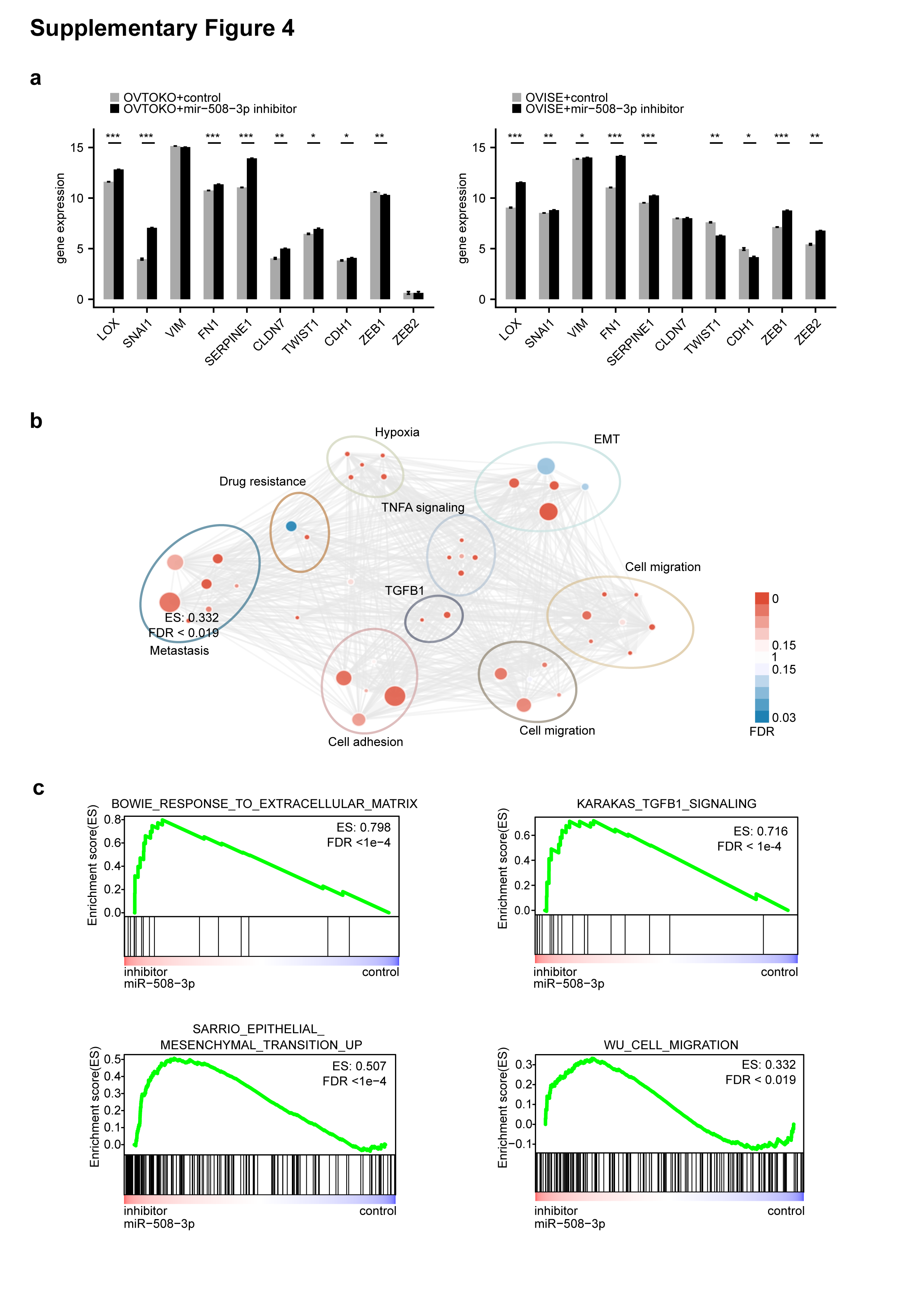

Supplement: Supplementary file 5 — Supplementary Fig.S4 [file 41388_2018_577_MOESM5_ESM.tif]

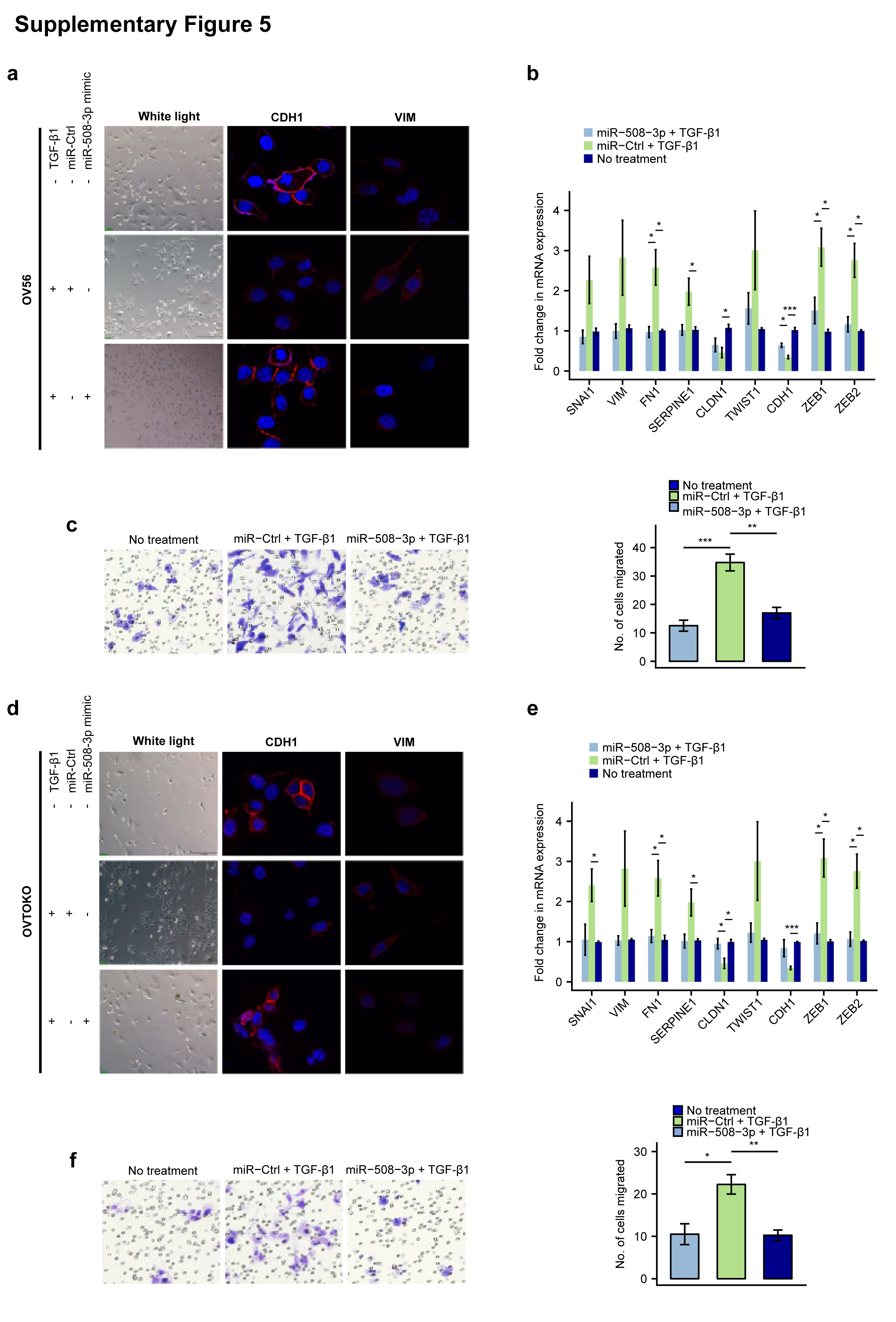

Supplement: Supplementary file 6 — Supplementary Fig.S5 [file 41388_2018_577_MOESM6_ESM.tif]

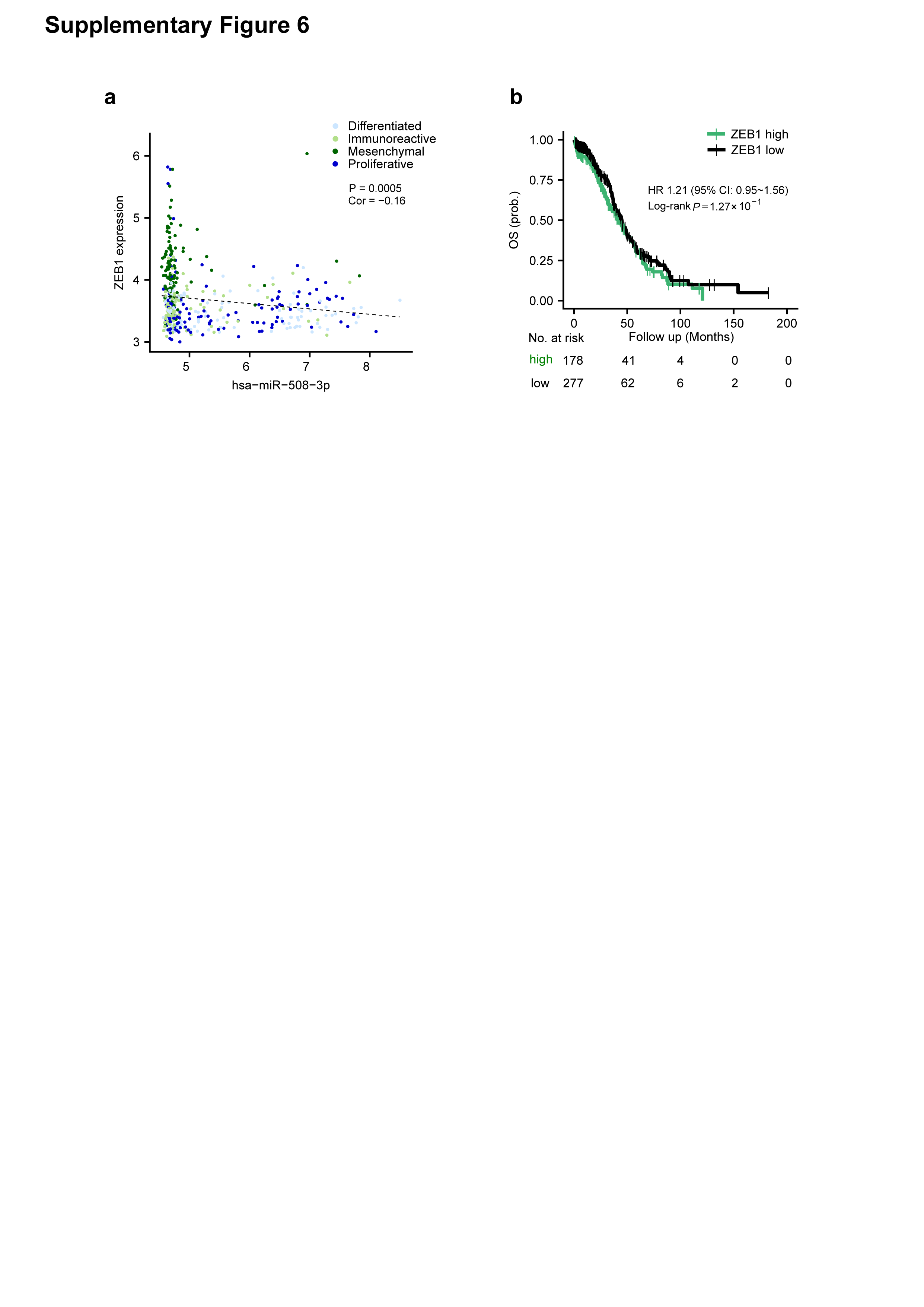

Supplement: Supplementary file 7 — Supplementary Fig.S6 [file 41388_2018_577_MOESM7_ESM.tif]

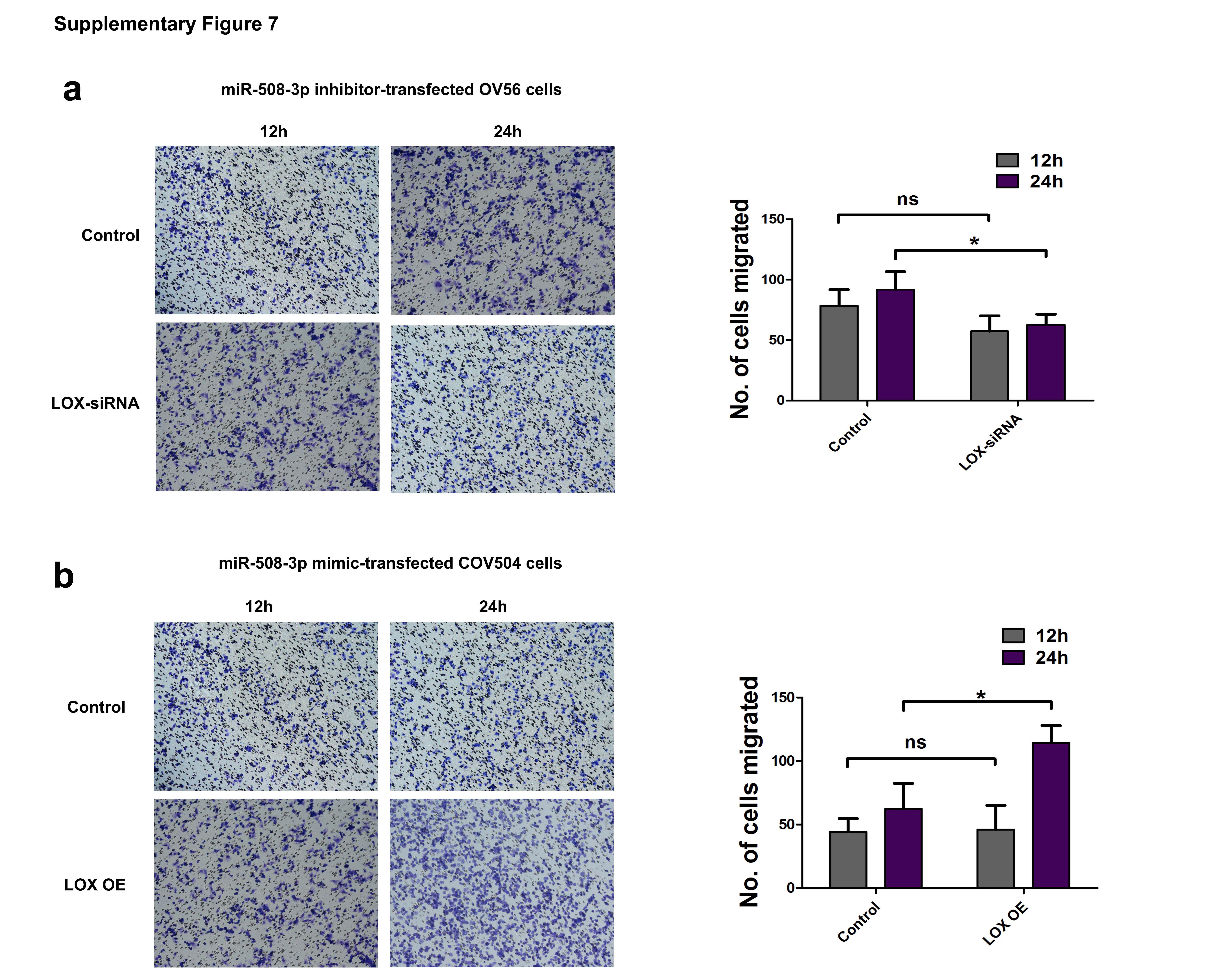

Supplement: Supplementary file 8 — Supplementary Fig.S7 [file 41388_2018_577_MOESM8_ESM.jpg]

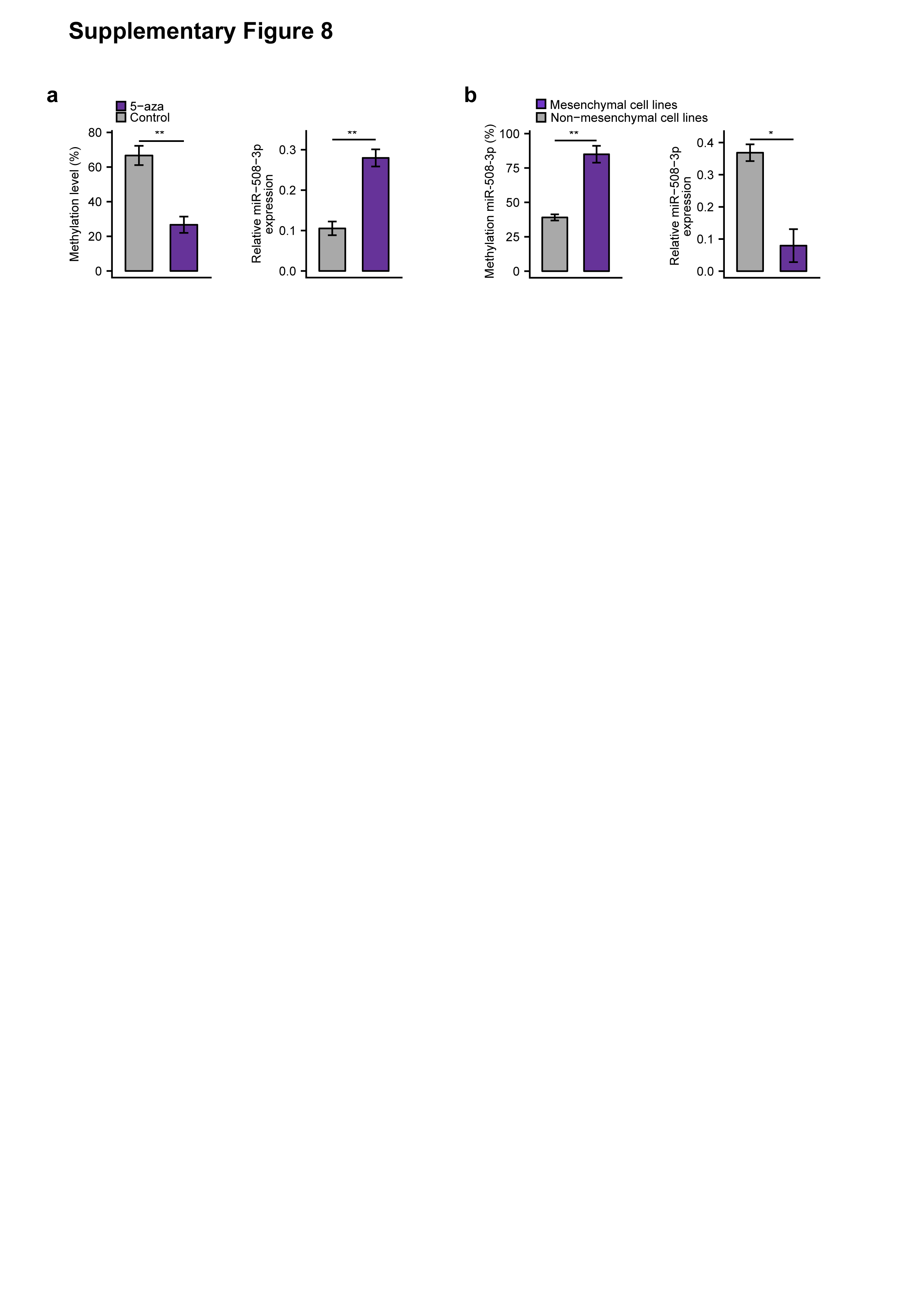

Supplement: Supplementary file 9 — Supplementary Fig.S8 [file 41388_2018_577_MOESM9_ESM.tif]
