# Supplementary material for: Integrative network biology analysis identifies miR-508-3p as the determinant for the mesenchymal identity and a strong prognostic biomarker of ovarian cancer
Source: Oncogene. 2018 Nov 26;38(13):2305–19. doi: 10.1038/s41388-018-0577-5 (PMC6755993; doi:10.1038/s41388-018-0577-5)
Supplement: Supplementary file 10 — Supplementary Table S1 [file 41388_2018_577_MOESM10_ESM.docx]

| **Supplementary Table S1. Overview of public datasets and the West China Cohort** | | |  |  |
| --- | --- | --- | --- | --- |
|  |  |  |  |  |
| **A** |  |  |  |  |
| **Patient data (miRNA)** | **Samples** | **Platform** | **Accession number** | **Reference** |
| The Cancer Genome Atlas (TCGA) Agilent | 537 | Agilent 244K Whole Genome Expression Array | http://cancergenome.nih.gov/ | The Cancer Genome Atlas Research Network, Nature, 2010 |
| OV179 | 179 | Agilent-035758 Human miRBASE 16.0 plus | GSE73581 | Marina et al. Lancet Oncol, 2016 |
| OV133 | 133 | Illumina Human v2 MicroRNA expression beadchip | GSE73582 | Marina et al. Lancet Oncol, 2016 |
| Bagnoli | 130 | Illumina Human v2 MicroRNA expression beadchip | GSE25204 | Bagnoli et al. Oncotarget, 2011 |
| Nam | 20 | Agilent-031181 Unrestricted_Human_miRNA_V16.0 | GSE83693 | Nam et al. Oncotarget, 2016 |
| **TOTAL** | **999** |  |  |  |
|  |  |  |  |  |
|  |  |  |  |  |
| **Patient data (mRNA)** | **Samples** | **Platform** | **Accession number** | **Reference** |
| The Cancer Genome Atlas (TCGA) Affymetrix | 490 | HG-U133A | http://cancergenome.nih.gov/ | The Cancer Genome Atlas Research Network, Nature, 2010 |
| Bonome | 182 | HG-U133A | GSE26712 | Bonome et al. Cancer Res, 2008 |
| Tothill | 285 | HG-U133_Plus_2 | GSE9891 | Tothill et al. Clin Cancer Res, 2008 |
| Mateescu | 107 | HG-U133_Plus_2 | GSE26193 | Mateescu et al. Nat Med, 2011 |
| **TOTAL** | **1064** |  |  |  |
|  |  |  |  |  |
|  |  |  |  |  |
| **Cell line data** | **Samples** | **Platform** | **Accession number** | **Reference** |
| Dezső | 189 | HG-U133_Plus_2 | GSE50831 | Dezső et al. PLoS One, 2014 |
|  |  |  |  |  |
| **B** |  |  |  |  |
| **Patient demographics and clinical characteristics** | |  |  |  |
|  | **West China Cohort** |  |  |  |
| **Number of patients** | **131** |  |  |  |
|  |  |  |  |  |
| Median age (year) | 52 |  |  |  |
| Median OS (month) | 34 |  |  |  |
| Stage |  |  |  |  |
| I | 12 |  |  |  |
| II | 14 |  |  |  |
| III | 75 |  |  |  |
| IV | 29 |  |  |  |
| na | 1 |  |  |  |
| Grade |  |  |  |  |
| 1 | 116 |  |  |  |
| 2 | 14 |  |  |  |
| na | 1 |  |  |  |
| Death |  |  |  |  |
| yes | 78 |  |  |  |
| no | 53 |  |  |  |
