# Supplementary material for: Integrative network biology analysis identifies miR-508-3p as the determinant for the mesenchymal identity and a strong prognostic biomarker of ovarian cancer
Source: Oncogene. 2018 Nov 26;38(13):2305–19. doi: 10.1038/s41388-018-0577-5 (PMC6755993; doi:10.1038/s41388-018-0577-5)
Supplement: Supplementary file 11 — Supplementary Table S2 [file 41388_2018_577_MOESM11_ESM.docx]

| **Supplementary Table S2. Ovarian cancer subtype mini-classifier (10 unique genes)** | | | | |  |
| --- | --- | --- | --- | --- | --- |
|  |  |  |  |  | |
|  | **Centroids** | |  |  | |
| **Gene** | **Mesenchymal** | **Non-mesenchymal** |  |  | |
| FBN1 | 1.22 | -0.31 |  |  | |
| SNAI2 | 1.22 | -0.31 |  |  | |
| CTSK | 1.20 | -0.31 |  |  | |
| SEPT11 | 1.17 | -0.30 |  |  | |
| COL5A2 | 1.08 | -0.28 |  |  | |
| LUM | 1.04 | -0.26 |  |  | |
| COL6A3 | 1.00 | -0.26 |  |  | |
| COL1A2 | 0.99 | -0.25 |  |  | |
| COL3A1 | 0.97 | -0.25 |  |  | |
| SPARC | 0.96 | -0.25 |  |  | |
