# Supplementary material for: Integrative network biology analysis identifies miR-508-3p as the determinant for the mesenchymal identity and a strong prognostic biomarker of ovarian cancer
Source: Oncogene. 2018 Nov 26;38(13):2305–19. doi: 10.1038/s41388-018-0577-5 (PMC6755993; doi:10.1038/s41388-018-0577-5)
Supplement: Supplementary file 12 — Supplementary Table S3 [file 41388_2018_577_MOESM12_ESM.docx]

| **Supplementary Table S3. Subtype information for samples in mRNA validation datasets** | | | | | | |
| --- | --- | --- | --- | --- | --- | --- |
|  |  |  |  |  |  |  |
| Tothill Dataset (GSE9891) | | |  | Mateescu Dataset (GSE26193) | | |
|  | **posterior probability** | |  |  | **posterior probability** | |
| ID | Mesenchymal | Non-mesenchymal |  | ID | Mesenchymal | Non-mesenchymal |
| GSM249714 | 0.02 | 0.98 |  | GSM642933 | 0.30 | 0.70 |
| GSM249715 | 0.00 | 1.00 |  | GSM642934 | 0.00 | 1.00 |
| GSM249716 | 0.00 | 1.00 |  | GSM642935 | 0.01 | 0.99 |
| GSM249717 | 0.11 | 0.89 |  | GSM642936 | 0.00 | 1.00 |
| GSM249718 | 0.05 | 0.95 |  | GSM642937 | 0.40 | 0.60 |
| GSM249719 | 0.03 | 0.97 |  | GSM642938 | 0.70 | 0.30 |
| GSM249720 | 0.05 | 0.95 |  | GSM642939 | 0.00 | 1.00 |
| GSM249721 | 0.05 | 0.95 |  | GSM642940 | 0.00 | 1.00 |
| GSM249722 | 0.00 | 1.00 |  | GSM642941 | 0.02 | 0.98 |
| GSM249723 | 0.04 | 0.96 |  | GSM642942 | 0.38 | 0.62 |
| GSM249724 | 0.02 | 0.98 |  | GSM642943 | 0.01 | 0.99 |
| GSM249725 | 0.11 | 0.89 |  | GSM642944 | 0.56 | 0.44 |
| GSM249726 | 0.11 | 0.89 |  | GSM642945 | 0.03 | 0.97 |
| GSM249727 | 0.02 | 0.98 |  | GSM642946 | 0.92 | 0.08 |
| GSM249728 | 0.11 | 0.89 |  | GSM642947 | 0.97 | 0.03 |
| GSM249729 | 0.03 | 0.97 |  | GSM642948 | 0.02 | 0.98 |
| GSM249730 | 0.06 | 0.94 |  | GSM642949 | 0.98 | 0.02 |
| GSM249731 | 0.00 | 1.00 |  | GSM642950 | 0.00 | 1.00 |
| GSM249732 | 0.03 | 0.97 |  | GSM642951 | 0.02 | 0.98 |
| GSM249733 | 0.39 | 0.61 |  | GSM642952 | 0.15 | 0.85 |
| GSM249734 | 0.06 | 0.94 |  | GSM642953 | 0.97 | 0.03 |
| GSM249735 | 0.61 | 0.39 |  | GSM642954 | 0.17 | 0.83 |
| GSM249736 | 0.03 | 0.97 |  | GSM642955 | 0.00 | 1.00 |
| GSM249737 | 0.90 | 0.10 |  | GSM642956 | 0.04 | 0.96 |
| GSM249738 | 0.00 | 1.00 |  | GSM642957 | 0.00 | 1.00 |
| GSM249739 | 0.77 | 0.23 |  | GSM642958 | 0.00 | 1.00 |
| GSM249740 | 0.91 | 0.09 |  | GSM642959 | 0.00 | 1.00 |
| GSM249741 | 0.88 | 0.12 |  | GSM642960 | 0.00 | 1.00 |
| GSM249742 | 0.95 | 0.05 |  | GSM642961 | 0.04 | 0.96 |
| GSM249743 | 0.97 | 0.03 |  | GSM642962 | 0.01 | 0.99 |
| GSM249744 | 0.00 | 1.00 |  | GSM642963 | 0.00 | 1.00 |
| GSM249745 | 0.75 | 0.25 |  | GSM642964 | 0.09 | 0.91 |
| GSM249746 | 0.00 | 1.00 |  | GSM642965 | 0.01 | 0.99 |
| GSM249747 | 0.28 | 0.72 |  | GSM642966 | 0.00 | 1.00 |
| GSM249748 | 0.97 | 0.03 |  | GSM642967 | 0.00 | 1.00 |
| GSM249749 | 0.02 | 0.98 |  | GSM642968 | 0.00 | 1.00 |
| GSM249750 | 1.00 | 0.00 |  | GSM642969 | 0.98 | 0.02 |
| GSM249751 | 0.84 | 0.16 |  | GSM642970 | 0.02 | 0.98 |
| GSM249752 | 0.90 | 0.10 |  | GSM642971 | 0.09 | 0.91 |
| GSM249753 | 0.01 | 0.99 |  | GSM642972 | 0.00 | 1.00 |
| GSM249754 | 0.00 | 1.00 |  | GSM642973 | 0.00 | 1.00 |
| GSM249755 | 0.50 | 0.50 |  | GSM642974 | 0.02 | 0.98 |
| GSM249756 | 0.01 | 0.99 |  | GSM642975 | 0.13 | 0.87 |
| GSM249757 | 0.51 | 0.49 |  | GSM642976 | 0.00 | 1.00 |
| GSM249758 | 0.00 | 1.00 |  | GSM642977 | 0.00 | 1.00 |
| GSM249759 | 0.96 | 0.04 |  | GSM642978 | 0.20 | 0.80 |
| GSM249760 | 0.01 | 0.99 |  | GSM642979 | 0.26 | 0.74 |
| GSM249761 | 0.96 | 0.04 |  | GSM642980 | 0.00 | 1.00 |
| GSM249762 | 0.25 | 0.75 |  | GSM642981 | 0.00 | 1.00 |
| GSM249763 | 0.00 | 1.00 |  | GSM642982 | 0.00 | 1.00 |
| GSM249764 | 0.97 | 0.03 |  | GSM642983 | 0.00 | 1.00 |
| GSM249765 | 0.00 | 1.00 |  | GSM642984 | 0.00 | 1.00 |
| GSM249766 | 0.00 | 1.00 |  | GSM642985 | 0.01 | 0.99 |
| GSM249767 | 0.15 | 0.85 |  | GSM642986 | 0.04 | 0.96 |
| GSM249768 | 0.44 | 0.56 |  | GSM642987 | 0.00 | 1.00 |
| GSM249769 | 0.00 | 1.00 |  | GSM642988 | 0.02 | 0.98 |
| GSM249770 | 0.00 | 1.00 |  | GSM642989 | 0.83 | 0.17 |
| GSM249771 | 0.78 | 0.22 |  | GSM642990 | 0.01 | 0.99 |
| GSM249773 | 0.19 | 0.81 |  | GSM642991 | 1.00 | 0.00 |
| GSM249774 | 0.00 | 1.00 |  | GSM642992 | 0.67 | 0.33 |
| GSM249775 | 0.00 | 1.00 |  | GSM642993 | 0.73 | 0.27 |
| GSM249776 | 0.00 | 1.00 |  | GSM642994 | 0.00 | 1.00 |
| GSM249777 | 0.00 | 1.00 |  | GSM642995 | 0.01 | 0.99 |
| GSM249778 | 0.00 | 1.00 |  | GSM642996 | 0.98 | 0.02 |
| GSM249779 | 0.00 | 1.00 |  | GSM642997 | 0.37 | 0.63 |
| GSM249780 | 0.51 | 0.49 |  | GSM642998 | 0.19 | 0.81 |
| GSM249781 | 0.26 | 0.74 |  | GSM642999 | 0.00 | 1.00 |
| GSM249782 | 0.89 | 0.11 |  | GSM643000 | 0.00 | 1.00 |
| GSM249783 | 0.00 | 1.00 |  | GSM643001 | 0.93 | 0.07 |
| GSM249784 | 0.09 | 0.91 |  | GSM643002 | 0.01 | 0.99 |
| GSM249785 | 0.00 | 1.00 |  | GSM643003 | 0.40 | 0.60 |
| GSM249786 | 0.00 | 1.00 |  | GSM643004 | 0.64 | 0.36 |
| GSM249788 | 0.00 | 1.00 |  | GSM643005 | 0.77 | 0.23 |
| GSM249789 | 0.02 | 0.98 |  | GSM643006 | 0.95 | 0.05 |
| GSM249790 | 0.60 | 0.40 |  | GSM643007 | 0.00 | 1.00 |
| GSM249791 | 0.00 | 1.00 |  | GSM643008 | 0.94 | 0.06 |
| GSM249792 | 0.00 | 1.00 |  | GSM643009 | 0.49 | 0.51 |
| GSM249793 | 0.01 | 0.99 |  | GSM643010 | 0.99 | 0.01 |
| GSM249794 | 0.00 | 1.00 |  | GSM643011 | 0.97 | 0.03 |
| GSM249795 | 0.83 | 0.17 |  | GSM643012 | 0.00 | 1.00 |
| GSM249796 | 0.02 | 0.98 |  | GSM643013 | 0.02 | 0.98 |
| GSM249797 | 0.92 | 0.08 |  | GSM643014 | 0.00 | 1.00 |
| GSM249798 | 0.10 | 0.90 |  | GSM643015 | 0.00 | 1.00 |
| GSM249799 | 0.00 | 1.00 |  | GSM643016 | 0.00 | 1.00 |
| GSM249800 | 0.00 | 1.00 |  | GSM643017 | 0.30 | 0.70 |
| GSM249801 | 0.05 | 0.95 |  | GSM643018 | 0.02 | 0.98 |
| GSM249802 | 0.00 | 1.00 |  | GSM643019 | 0.00 | 1.00 |
| GSM249803 | 0.51 | 0.49 |  | GSM643020 | 0.25 | 0.75 |
| GSM249804 | 0.00 | 1.00 |  | GSM643021 | 0.01 | 0.99 |
| GSM249805 | 0.20 | 0.80 |  | GSM643022 | 0.00 | 1.00 |
| GSM249806 | 0.91 | 0.09 |  | GSM643023 | 0.00 | 1.00 |
| GSM249807 | 0.01 | 0.99 |  | GSM643024 | 0.65 | 0.35 |
| GSM249808 | 0.01 | 0.99 |  | GSM643025 | 0.15 | 0.85 |
| GSM249809 | 0.57 | 0.43 |  | GSM643026 | 0.00 | 1.00 |
| GSM249810 | 0.67 | 0.33 |  | GSM643027 | 0.90 | 0.10 |
| GSM249811 | 0.15 | 0.85 |  | GSM643028 | 0.00 | 1.00 |
| GSM249812 | 0.00 | 1.00 |  | GSM643029 | 0.15 | 0.85 |
| GSM249813 | 0.47 | 0.53 |  | GSM643030 | 0.10 | 0.90 |
| GSM249814 | 0.84 | 0.16 |  | GSM643031 | 0.00 | 1.00 |
| GSM249815 | 0.14 | 0.86 |  | GSM643032 | 0.95 | 0.05 |
| GSM249816 | 0.54 | 0.46 |  | GSM643033 | 0.03 | 0.97 |
| GSM249817 | 0.89 | 0.11 |  | GSM643034 | 0.09 | 0.91 |
| GSM249818 | 0.31 | 0.69 |  | GSM643035 | 0.99 | 0.01 |
| GSM249819 | 0.00 | 1.00 |  | GSM643036 | 0.00 | 1.00 |
| GSM249820 | 0.06 | 0.94 |  | GSM643037 | 0.17 | 0.83 |
| GSM249821 | 0.99 | 0.01 |  | GSM643038 | 0.30 | 0.70 |
| GSM249822 | 0.01 | 0.99 |  | GSM643039 | 0.81 | 0.19 |
| GSM249823 | 0.42 | 0.58 |  |  |  |  |
| GSM249824 | 0.03 | 0.97 |  |  |  |  |
| GSM249825 | 0.48 | 0.52 |  |  |  |  |
| GSM249826 | 0.01 | 0.99 |  |  |  |  |
| GSM249827 | 0.12 | 0.88 |  |  |  |  |
| GSM249828 | 0.03 | 0.97 |  |  |  |  |
| GSM249829 | 0.85 | 0.15 |  |  |  |  |
| GSM249830 | 0.07 | 0.93 |  |  |  |  |
| GSM249831 | 0.64 | 0.36 |  |  |  |  |
| GSM249832 | 0.00 | 1.00 |  |  |  |  |
| GSM249833 | 0.01 | 0.99 |  |  |  |  |
| GSM249834 | 0.71 | 0.29 |  |  |  |  |
| GSM249835 | 0.68 | 0.32 |  |  |  |  |
| GSM249836 | 0.99 | 0.01 |  |  |  |  |
| GSM249837 | 0.00 | 1.00 |  |  |  |  |
| GSM249838 | 0.86 | 0.14 |  |  |  |  |
| GSM249839 | 0.03 | 0.97 |  |  |  |  |
| GSM249840 | 0.40 | 0.60 |  |  |  |  |
| GSM249841 | 0.00 | 1.00 |  |  |  |  |
| GSM249842 | 0.01 | 0.99 |  |  |  |  |
| GSM249843 | 0.73 | 0.27 |  |  |  |  |
| GSM249844 | 0.00 | 1.00 |  |  |  |  |
| GSM249845 | 0.97 | 0.03 |  |  |  |  |
| GSM249846 | 0.94 | 0.06 |  |  |  |  |
| GSM249847 | 0.00 | 1.00 |  |  |  |  |
| GSM249848 | 0.87 | 0.13 |  |  |  |  |
| GSM249849 | 0.02 | 0.98 |  |  |  |  |
| GSM249850 | 0.00 | 1.00 |  |  |  |  |
| GSM249851 | 0.01 | 0.99 |  |  |  |  |
| GSM249852 | 0.03 | 0.97 |  |  |  |  |
| GSM249853 | 0.08 | 0.92 |  |  |  |  |
| GSM249854 | 0.92 | 0.08 |  |  |  |  |
| GSM249855 | 0.00 | 1.00 |  |  |  |  |
| GSM249856 | 0.00 | 1.00 |  |  |  |  |
| GSM249857 | 0.06 | 0.94 |  |  |  |  |
| GSM249858 | 1.00 | 0.00 |  |  |  |  |
| GSM249859 | 0.63 | 0.37 |  |  |  |  |
| GSM249860 | 0.92 | 0.08 |  |  |  |  |
| GSM249861 | 0.00 | 1.00 |  |  |  |  |
| GSM249862 | 0.19 | 0.81 |  |  |  |  |
| GSM249863 | 0.82 | 0.18 |  |  |  |  |
| GSM249864 | 0.00 | 1.00 |  |  |  |  |
| GSM249865 | 0.45 | 0.55 |  |  |  |  |
| GSM249866 | 0.00 | 1.00 |  |  |  |  |
| GSM249867 | 0.88 | 0.12 |  |  |  |  |
| GSM249868 | 0.00 | 1.00 |  |  |  |  |
| GSM249869 | 0.04 | 0.96 |  |  |  |  |
| GSM249870 | 0.00 | 1.00 |  |  |  |  |
| GSM249871 | 0.87 | 0.13 |  |  |  |  |
| GSM249872 | 0.92 | 0.08 |  |  |  |  |
| GSM249873 | 0.51 | 0.49 |  |  |  |  |
| GSM249874 | 0.06 | 0.94 |  |  |  |  |
| GSM249875 | 0.07 | 0.93 |  |  |  |  |
| GSM249876 | 0.87 | 0.13 |  |  |  |  |
| GSM249877 | 0.02 | 0.98 |  |  |  |  |
| GSM249878 | 0.00 | 1.00 |  |  |  |  |
| GSM249879 | 0.96 | 0.04 |  |  |  |  |
| GSM249880 | 0.00 | 1.00 |  |  |  |  |
| GSM249881 | 0.00 | 1.00 |  |  |  |  |
| GSM249882 | 0.03 | 0.97 |  |  |  |  |
| GSM249883 | 0.00 | 1.00 |  |  |  |  |
| GSM249884 | 0.00 | 1.00 |  |  |  |  |
| GSM249885 | 0.49 | 0.51 |  |  |  |  |
| GSM249886 | 0.00 | 1.00 |  |  |  |  |
| GSM249887 | 0.07 | 0.93 |  |  |  |  |
| GSM249888 | 0.84 | 0.16 |  |  |  |  |
| GSM249889 | 0.01 | 0.99 |  |  |  |  |
| GSM249890 | 0.90 | 0.10 |  |  |  |  |
| GSM249891 | 0.01 | 0.99 |  |  |  |  |
| GSM249892 | 0.00 | 1.00 |  |  |  |  |
| GSM249893 | 0.00 | 1.00 |  |  |  |  |
| GSM249894 | 0.00 | 1.00 |  |  |  |  |
| GSM249895 | 0.97 | 0.03 |  |  |  |  |
| GSM249896 | 0.98 | 0.02 |  |  |  |  |
| GSM249897 | 0.00 | 1.00 |  |  |  |  |
| GSM249898 | 0.01 | 0.99 |  |  |  |  |
| GSM249899 | 0.01 | 0.99 |  |  |  |  |
| GSM249900 | 0.00 | 1.00 |  |  |  |  |
| GSM249901 | 0.00 | 1.00 |  |  |  |  |
| GSM249902 | 0.01 | 0.99 |  |  |  |  |
| GSM249903 | 0.19 | 0.81 |  |  |  |  |
| GSM249904 | 0.00 | 1.00 |  |  |  |  |
| GSM249905 | 0.00 | 1.00 |  |  |  |  |
| GSM249906 | 0.05 | 0.95 |  |  |  |  |
| GSM249907 | 0.55 | 0.45 |  |  |  |  |
| GSM249908 | 0.06 | 0.94 |  |  |  |  |
| GSM249909 | 0.98 | 0.02 |  |  |  |  |
| GSM249910 | 0.07 | 0.93 |  |  |  |  |
| GSM249911 | 0.99 | 0.01 |  |  |  |  |
| GSM249912 | 0.00 | 1.00 |  |  |  |  |
| GSM249913 | 0.00 | 1.00 |  |  |  |  |
| GSM249914 | 0.75 | 0.25 |  |  |  |  |
| GSM249915 | 0.94 | 0.06 |  |  |  |  |
| GSM249916 | 0.00 | 1.00 |  |  |  |  |
| GSM249917 | 0.77 | 0.23 |  |  |  |  |
| GSM249918 | 0.00 | 1.00 |  |  |  |  |
| GSM249919 | 0.13 | 0.87 |  |  |  |  |
| GSM249920 | 0.22 | 0.78 |  |  |  |  |
| GSM249921 | 0.00 | 1.00 |  |  |  |  |
| GSM249922 | 0.00 | 1.00 |  |  |  |  |
| GSM249923 | 0.88 | 0.12 |  |  |  |  |
| GSM249924 | 0.03 | 0.97 |  |  |  |  |
| GSM249925 | 0.30 | 0.70 |  |  |  |  |
| GSM249926 | 0.00 | 1.00 |  |  |  |  |
| GSM249927 | 0.29 | 0.71 |  |  |  |  |
| GSM249928 | 0.00 | 1.00 |  |  |  |  |
| GSM249929 | 0.00 | 1.00 |  |  |  |  |
| GSM249930 | 0.00 | 1.00 |  |  |  |  |
| GSM249931 | 0.95 | 0.05 |  |  |  |  |
| GSM249932 | 0.14 | 0.86 |  |  |  |  |
| GSM249933 | 0.01 | 0.99 |  |  |  |  |
| GSM249934 | 0.01 | 0.99 |  |  |  |  |
| GSM249935 | 0.61 | 0.39 |  |  |  |  |
| GSM249936 | 0.00 | 1.00 |  |  |  |  |
| GSM249937 | 0.00 | 1.00 |  |  |  |  |
| GSM249938 | 0.00 | 1.00 |  |  |  |  |
| GSM249939 | 0.00 | 1.00 |  |  |  |  |
| GSM249940 | 0.39 | 0.61 |  |  |  |  |
| GSM249941 | 0.18 | 0.82 |  |  |  |  |
| GSM249942 | 0.00 | 1.00 |  |  |  |  |
| GSM249943 | 0.01 | 0.99 |  |  |  |  |
| GSM249944 | 0.00 | 1.00 |  |  |  |  |
| GSM249945 | 0.65 | 0.35 |  |  |  |  |
| GSM249946 | 0.43 | 0.57 |  |  |  |  |
| GSM249947 | 0.98 | 0.02 |  |  |  |  |
| GSM249948 | 0.01 | 0.99 |  |  |  |  |
| GSM249949 | 0.00 | 1.00 |  |  |  |  |
| GSM249950 | 0.23 | 0.77 |  |  |  |  |
| GSM249951 | 0.82 | 0.18 |  |  |  |  |
| GSM249953 | 0.00 | 1.00 |  |  |  |  |
| GSM249954 | 0.00 | 1.00 |  |  |  |  |
| GSM249955 | 0.08 | 0.92 |  |  |  |  |
| GSM249956 | 0.77 | 0.23 |  |  |  |  |
| GSM249957 | 0.38 | 0.62 |  |  |  |  |
| GSM249958 | 0.00 | 1.00 |  |  |  |  |
| GSM249959 | 0.00 | 1.00 |  |  |  |  |
| GSM249960 | 0.00 | 1.00 |  |  |  |  |
| GSM249961 | 0.00 | 1.00 |  |  |  |  |
| GSM249962 | 0.10 | 0.90 |  |  |  |  |
| GSM249963 | 0.02 | 0.98 |  |  |  |  |
| GSM249964 | 0.49 | 0.51 |  |  |  |  |
| GSM249965 | 0.01 | 0.99 |  |  |  |  |
| GSM249966 | 0.00 | 1.00 |  |  |  |  |
| GSM249967 | 0.00 | 1.00 |  |  |  |  |
| GSM249968 | 0.00 | 1.00 |  |  |  |  |
| GSM249969 | 0.17 | 0.83 |  |  |  |  |
| GSM249970 | 0.47 | 0.53 |  |  |  |  |
| GSM249971 | 0.00 | 1.00 |  |  |  |  |
| GSM249972 | 0.01 | 0.99 |  |  |  |  |
| GSM249973 | 0.00 | 1.00 |  |  |  |  |
| GSM249974 | 0.00 | 1.00 |  |  |  |  |
| GSM249975 | 0.09 | 0.91 |  |  |  |  |
| GSM249976 | 0.00 | 1.00 |  |  |  |  |
| GSM249977 | 0.08 | 0.92 |  |  |  |  |
| GSM249978 | 0.96 | 0.04 |  |  |  |  |
| GSM249979 | 0.71 | 0.29 |  |  |  |  |
| GSM249980 | 0.00 | 1.00 |  |  |  |  |
| GSM249981 | 0.00 | 1.00 |  |  |  |  |
| GSM249982 | 0.85 | 0.15 |  |  |  |  |
| GSM249983 | 0.02 | 0.98 |  |  |  |  |
| GSM249984 | 0.23 | 0.77 |  |  |  |  |
| GSM249985 | 0.04 | 0.96 |  |  |  |  |
| GSM249986 | 0.02 | 0.98 |  |  |  |  |
| GSM249987 | 0.02 | 0.98 |  |  |  |  |
| GSM249988 | 0.78 | 0.22 |  |  |  |  |
| GSM249989 | 0.89 | 0.11 |  |  |  |  |
| GSM249990 | 0.14 | 0.86 |  |  |  |  |
| GSM249991 | 0.00 | 1.00 |  |  |  |  |
| GSM249992 | 0.97 | 0.03 |  |  |  |  |
| GSM249993 | 0.01 | 0.99 |  |  |  |  |
| GSM249994 | 0.05 | 0.95 |  |  |  |  |
| GSM249995 | 0.00 | 1.00 |  |  |  |  |
| GSM249996 | 0.00 | 1.00 |  |  |  |  |
| GSM249997 | 0.98 | 0.02 |  |  |  |  |
| GSM249998 | 0.00 | 1.00 |  |  |  |  |
| GSM249999 | 0.00 | 1.00 |  |  |  |  |
| GSM250000 | 0.08 | 0.92 |  |  |  |  |
| GSM250001 | 0.52 | 0.48 |  |  |  |  |
