# Supplementary material for: Integrative network biology analysis identifies miR-508-3p as the determinant for the mesenchymal identity and a strong prognostic biomarker of ovarian cancer
Source: Oncogene. 2018 Nov 26;38(13):2305–19. doi: 10.1038/s41388-018-0577-5 (PMC6755993; doi:10.1038/s41388-018-0577-5)
Supplement: Supplementary file 13 — Supplementary Table S4 [file 41388_2018_577_MOESM13_ESM.docx]

| **Supplementary Table S4. Clinical Information and PCR expression in West China Cohort** | | | | | | | | |  |  |
| --- | --- | --- | --- | --- | --- | --- | --- | --- | --- | --- |
|  |  |  |  |  |  |  |  |  | |  |
| Patients ID | Age | Stage | Grade | Vital Status (0=Alive; 1=Dead) | OS (months) | has-miR-508-3p | LOX |  |  |  |
| patients_1 | 53 | Ib | 1 | 0 | 97 | 2.20 | 0.33 |  |  |  |
| patients_2 | 57 | IV | 2 | 0 | 133 | 6.95 | 2.26 |  |  |  |
| patients_3 | 49 | III | 1 | 1 | 50 | 1.00 | 0.13 |  |  |  |
| patients_4 | 70 | Ia | 1 | 1 | 36 | 0.04 | 4.25 |  |  |  |
| patients_5 | 49 | IIIc | 1 | 0 | 129 | 3.89 | 1.40 |  |  |  |
| patients_6 | 59 | II | 2 | 0 | 128 | 2.85 | 2.38 |  |  |  |
| patients_7 | 55 | IIIc | 2 | 1 | 50 | 3.00 | 4.91 |  |  |  |
| patients_8 | 51 | IIIc | 2 | 1 | 108 | 5.59 | 1.10 |  |  |  |
| patients_9 | 52 | IIIc | 1 | 1 | 49 | 0.00 | 5.23 |  |  |  |
| patients_10 | 48 | II | 2 | 1 | 108 | 2.62 | 0.19 |  |  |  |
| patients_11 | 50 | III | 2 | 1 | 53 | 1.84 | 4.08 |  |  |  |
| patients_12 | 55 | III | 1 | 1 | 14 | 0.01 | 8.86 |  |  |  |
| patients_13 | 52 | IIIc | 1 | 0 | 126 | 6.30 | 3.55 |  |  |  |
| patients_14 | 36 | III | 1 | 1 | 47 | 1.10 | 3.65 |  |  |  |
| patients_15 | 42 | IIIc | 1 | 1 | 7 | 0.00 | 7.01 |  |  |  |
| patients_16 | 54 | IIc | 1 | 1 | 16 | 0.00 | 7.00 |  |  |  |
| patients_17 | 44 | II | 2 | 1 | 101 | 1.66 | 2.96 |  |  |  |
| patients_18 | 47 | III | 1 | 1 | 44 | 0.00 | 3.00 |  |  |  |
| patients_19 | 45 | IIIc | 1 | 1 | 14 | 0.09 | 0.07 |  |  |  |
| patients_20 | 60 | IIIc | 1 | 1 | 25 | 0.10 | 6.55 |  |  |  |
| patients_21 | 56 | III | 1 | 1 | 16 | 2.00 | 4.79 |  |  |  |
| patients_22 | 43 | IIIc | 2 | 1 | 48 | 0.00 | 3.36 |  |  |  |
| patients_23 | 45 | III | 1 | 1 | 32 | 0.00 | 6.65 |  |  |  |
| patients_24 | 45 | II | 1 | 1 | 43 | 1.28 | 3.77 |  |  |  |
| patients_25 | 61 | II | 1 | 1 | 47 | 0.00 | 0.23 |  |  |  |
| patients_26 | 47 | III | 1 | 0 | 111 | 5.93 | 0.61 |  |  |  |
| patients_27 | 62 | I | 1 | 1 | 35 | 0.01 | 7.00 |  |  |  |
| patients_28 | 50 | I | 1 | 0 | 110 | 3.92 | 1.99 |  |  |  |
| patients_29 | 52 | IV | 1 | 1 | 70 | 0.20 | 2.72 |  |  |  |
| patients_30 | 69 | III | 1 | 1 | 34 | 0.01 | 7.31 |  |  |  |
| patients_31 | 42 | IIIc | 1 | 1 | 15 | 0.00 | 6.02 |  |  |  |
| patients_32 | 55 | IIIc | 1 | 1 | 14 | 0.00 | 4.21 |  |  |  |
| patients_33 | 68 | IIIc | 1 | 1 | 6 | 0.00 | 9.08 |  |  |  |
| patients_34 | 60 | Ic | 1 | 1 | 29 | 0.01 | 4.28 |  |  |  |
| patients_35 | 43 | IIIc | 2 | 1 | 8 | 0.00 | 8.01 |  |  |  |
| patients_36 | 64 | IIIc | 1 | 1 | 40 | 0.18 | 1.29 |  |  |  |
| patients_37 | 50 | II | 1 | 1 | 60 | 2.04 | 0.05 |  |  |  |
| patients_38 | 42 | II | 1 | 1 | 39 | 1.29 | 6.33 |  |  |  |
| patients_39 | 37 | IIIC | 1 | 1 | 30 | 1.98 | 4.20 |  |  |  |
| patients_40 | 54 | IIIc | 1 | 1 | 25 | 0.21 | 7.92 |  |  |  |
| patients_41 | 50 | IV | 1 | 1 | 41 | 0.00 | 2.31 |  |  |  |
| patients_42 | 63 | IIIc | 1 | 0 | 88 | 3.50 | 2.38 |  |  |  |
| patients_43 | 61 | III | 2 | 1 | 34 | 0.03 | 0.16 |  |  |  |
| patients_44 | 61 | II | 2 | 1 | 86 | 0.01 | 2.28 |  |  |  |
| patients_45 | 40 | III | 1 | 1 | 21 | 0.03 | 7.21 |  |  |  |
| patients_46 | 45 | III | 1 | 1 | 37 | 2.57 | 0.05 |  |  |  |
| patients_47 | 69 | III | 1 | 1 | 34 | 0.20 | 5.74 |  |  |  |
| patients_48 | 68 | II | 1 | 1 | 84 | 4.97 | 2.89 |  |  |  |
| patients_49 | 60 | III | 1 | 1 | 19 | 0.02 | 4.28 |  |  |  |
| patients_50 | 56 | IIIc | 1 | 0 | 118 | 6.00 | 2.22 |  |  |  |
| patients_51 | 56 | III | 1 | 1 | 24 | 2.01 | 3.68 |  |  |  |
| patients_52 | 38 | IV | 1 | 1 | 26 | 0.01 | 0.16 |  |  |  |
| patients_53 | 54 | IV | 2 | 1 | 50 | 0.90 | 0.13 |  |  |  |
| patients_54 | 61 | IIc | 1 | 1 | 17 | 0.00 | 8.18 |  |  |  |
| patients_55 | 61 | IIb | 1 | 1 | 25 | 0.24 | 1.44 |  |  |  |
| patients_56 | 56 | IIIc | 1 | 1 | 14 | 0.00 | 9.01 |  |  |  |
| patients_57 | 42 | IIIc | 1 | 1 | 14 | 0.00 | 6.93 |  |  |  |
| patients_58 | 53 | III | 1 | 0 | 41 | 1.94 | 2.59 |  |  |  |
| patients_59 | 53 | III | 1 | 0 | 77 | 3.01 | 0.59 |  |  |  |
| patients_60 | 42 | IV | 1 | 1 | 4 | 0.00 | 7.01 |  |  |  |
| patients_61 | 70 | IV | 1 | 1 | 6 | 0.00 | 0.77 |  |  |  |
| patients_62 | 59 | III | 1 | 0 | 15 | 0.00 | 6.09 |  |  |  |
| patients_63 | 64 | III | 1 | 1 | 20 | 0.04 | 7.13 |  |  |  |
| patients_64 | 61 | IIIc | 1 | 0 | 62 | 2.19 | 3.21 |  |  |  |
| patients_65 | 39 | IIIc | 1 | 1 | 9 | 0.00 | 8.12 |  |  |  |
| patients_66 | 58 | IV | 1 | 1 | 7 | 0.00 | 6.21 |  |  |  |
| patients_67 | 42 | IIIc | 1 | 0 | 70 | 3.99 | 1.90 |  |  |  |
| patients_68 | 70 | IIIc | 1 | 0 | 46 | 0.85 | 3.09 |  |  |  |
| patients_69 | 57 | III | 1 | 1 | 18 | 0.00 | 5.03 |  |  |  |
| patients_70 | 44 | III | 1 | 1 | 31 | 0.01 | 2.17 |  |  |  |
| patients_71 | 61 | III | 1 | 1 | 31 | 2.87 | 1.04 |  |  |  |
| patients_72 | 52 | IIIc | 1 | 0 | 31 | 0.00 | 4.08 |  |  |  |
| patients_73 | 48 | IIIc | 1 | 0 | 34 | 0.00 | 2.95 |  |  |  |
| patients_74 | 42 | Ib | 1 | 1 | 15 | 0.00 | 4.73 |  |  |  |
| patients_75 | 50 | IIIc | 1 | 0 | 57 | 2.00 | 0.24 |  |  |  |
| patients_76 | 62 | IIIc | 1 | 1 | 16 | 0.00 | 0.31 |  |  |  |
| patients_77 | 52 | Ib | 1 | 1 | 14 | 0.00 | 5.19 |  |  |  |
| patients_78 | 71 | III | 1 | 1 | 21 | 0.11 | 5.00 |  |  |  |
| patients_79 | 61 | IIIb | 1 | 0 | 70 | 3.00 | 2.03 |  |  |  |
| patients_80 | 57 | IV | 1 | 1 | 16 | 0.00 | 5.01 |  |  |  |
| patients_81 | 49 | III | 1 | 0 | 56 | 2.69 | 2.38 |  |  |  |
| patients_82 | 54 | IIIc | 1 | 0 | 65 | 2.93 | 2.24 |  |  |  |
| patients_83 | 50 | III | 1 | 1 | 12 | 0.02 | 9.74 |  |  |  |
| patients_84 | 50 | IV | 1 | 1 | 24 | 1.00 | 6.82 |  |  |  |
| patients_85 | 45 | IV | 1 | 1 | 16 | 0.00 | 5.01 |  |  |  |
| patients_86 | 46 | IIIc | 1 | 1 | 24 | 0.00 | 6.09 |  |  |  |
| patients_87 | 42 | IIIc |  | 1 | 25 | 0.00 | 5.01 |  |  |  |
| patients_88 | 61 |  | 1 | 0 | 11 | 0.00 | 6.58 |  |  |  |
| patients_89 | 50 | I | 1 | 0 | 64 | 2.11 | 3.33 |  |  |  |
| patients_90 | 57 | IIIc | 1 | 0 | 5 | 0.00 | 2.62 |  |  |  |
| patients_91 | 59 | I | 1 | 0 | 26 | 0.00 | 7.94 |  |  |  |
| patients_92 | 59 | I | 1 | 0 | 47 | 1.95 | 1.57 |  |  |  |
| patients_93 | 39 | IIIc | 1 | 1 | 10 | 0.00 | 0.11 |  |  |  |
| patients_94 | 45 | IIIc | 1 | 0 | 50 | 2.97 | 4.21 |  |  |  |
| patients_95 | 41 | III | 1 | 1 | 11 | 2.00 | 8.05 |  |  |  |
| patients_96 | 48 | II | 1 | 0 | 14 | 0.00 | 7.10 |  |  |  |
| patients_97 | 44 | III | 1 | 1 | 18 | 0.00 | 6.14 |  |  |  |
| patients_98 | 44 | III | 1 | 1 | 21 | 0.02 | 1.10 |  |  |  |
| patients_99 | 43 | Ic | 1 | 1 | 16 | 0.00 | 9.09 |  |  |  |
| patients_100 | 39 | IIIc | 1 | 1 | 16 | 0.45 | 5.84 |  |  |  |
| patients_101 | 54 | IIIc | 1 | 0 | 48 | 0.84 | 9.88 |  |  |  |
| patients_102 | 59 | III | 1 | 0 | 9 | 0.00 | 8.01 |  |  |  |
| patients_103 | 54 | I | 1 | 0 | 130 | 4.85 | 0.22 |  |  |  |
| patients_104 | 40 | III | 1 | 0 | 15 | 0.00 | 0.13 |  |  |  |
| patients_105 | 49 | III | 1 | 0 | 80 | 1.28 | 3.41 |  |  |  |
| patients_106 | 59 | III | 1 | 0 | 60 | 3.94 | 3.27 |  |  |  |
| patients_107 | 59 | IIIc | 1 | 0 | 12 | 1.90 | 6.20 |  |  |  |
| patients_108 | 49 | III | 1 | 0 | 61 | 2.31 | 3.09 |  |  |  |
| patients_109 | 48 | IIIc | 1 | 0 | 74 | 0.29 | 2.33 |  |  |  |
| patients_110 | 44 | IIA | 1 | 0 | 64 | 3.63 | 2.79 |  |  |  |
| patients_111 | 55 | IIIc | 1 | 0 | 12 | 0.00 | 7.83 |  |  |  |
| patients_112 | 60 | IIIC | 1 | 0 | 28 | 0.08 | 6.74 |  |  |  |
| patients_113 | 64 | IIIc | 1 | 0 | 5 | 0.00 | 8.03 |  |  |  |
| patients_114 | 38 | IV | 2 | 1 | 50 | 2.84 | 4.83 |  |  |  |
| patients_115 | 49 | IV | 1 | 1 | 1 | 0.00 | 5.10 |  |  |  |
| patients_116 | 54 | IV | 1 | 0 | 105 | 3.73 | 1.00 |  |  |  |
| patients_117 | 58 | IV | 1 | 1 | 15 | 0.00 | 2.54 |  |  |  |
| patients_118 | 51 | IV | 1 | 0 | 40 | 2.95 | 2.64 |  |  |  |
| patients_119 | 47 | IV | 1 | 0 | 65 | 2.02 | 2.07 |  |  |  |
| patients_120 | 60 | IV | 1 | 0 | 35 | 0.08 | 7.59 |  |  |  |
| patients_121 | 51 | IV | 1 | 1 | 20 | 1.01 | 3.09 |  |  |  |
| patients_122 | 45 | IV | 1 | 0 | 50 | 3.20 | 2.60 |  |  |  |
| patients_123 | 73 | IV | 1 | 1 | 13 | 0.03 | 7.02 |  |  |  |
| patients_124 | 51 | IV | 1 | 1 | 19 | 2.55 | 0.94 |  |  |  |
| patients_125 | 44 | IV | 2 | 0 | 23 | 0.10 | 0.40 |  |  |  |
| patients_126 | 50 | IV | 1 | 0 | 47 | 1.49 | 0.10 |  |  |  |
| patients_127 | 55 | IV | 1 | 0 | 42 | 0.01 | 3.01 |  |  |  |
| patients_128 | 40 | IV | 1 | 0 | 42 | 1.75 | 2.39 |  |  |  |
| patients_129 | 62 | IV | 1 | 0 | 29 | 0.01 | 1.64 |  |  |  |
| patients_130 | 62 | IV | 1 | 0 | 41 | 2.77 | 2.04 |  |  |  |
| patients_131 | 44 | IV | 1 | 0 | 39 | 1.05 | 4.06 |  |  |  |
