# Supplementary material for: Integrative network biology analysis identifies miR-508-3p as the determinant for the mesenchymal identity and a strong prognostic biomarker of ovarian cancer
Source: Oncogene. 2018 Nov 26;38(13):2305–19. doi: 10.1038/s41388-018-0577-5 (PMC6755993; doi:10.1038/s41388-018-0577-5)
Supplement: Supplementary file 14 — Supplementary Table S5 [file 41388_2018_577_MOESM14_ESM.docx]

| **Supplementary Table S5. Univariate analysis of ovarall survival** | |  |
| --- | --- | --- |
|  |  |  |
|  | **Univariate analysis** | |
|  | HR (95% CI) | p value |
| **OV133 (n=133, events=51)** |  |  |
| has-miR-508-3p (low vs high) | 1.98 (1.09~3.59) | 0.025 |
| **OV179 (n=179, events=77)** |  |  |
| has-miR-508-3p (low vs high) | 1.99 (1.13~3.51) | 0.017 |
| **Mateescu (n=130, events=54)** |  |  |
| has-miR-508-3p (low vs high) | 3.70 (2.08~6.60) | <0·0001 |
