# Supplementary material for: Integrative network biology analysis identifies miR-508-3p as the determinant for the mesenchymal identity and a strong prognostic biomarker of ovarian cancer
Source: Oncogene. 2018 Nov 26;38(13):2305–19. doi: 10.1038/s41388-018-0577-5 (PMC6755993; doi:10.1038/s41388-018-0577-5)
Supplement: Supplementary file 15 — Supplementary Table S6 [file 41388_2018_577_MOESM15_ESM.docx]

| **Supplementary Table S6. Cox proportional hazards model for West China Cohort** | | | | | |
| --- | --- | --- | --- | --- | --- |
|  |  |  |  |  |  |
|  | **Univariate analysis** | |  | **Multivariable analysis** | |
|  | HR (95% CI) | p value |  | HR (95% CI) | p value |
| Age (>=65 vs <65) | 2.09 (1.00~4.36) | 0.05 |  | 1.36 (0.64~2.88) | 0.43 |
| stage (III–IV vs I–II) | 1.25 (0.73~2.15) | 0.41 |  | 1.01 (0.58~1.78) | 0.96 |
| grade (2 vs 1) | 0.78 (0.41~1.50) | 0.46 |  | 0.82 (0.41~1.61) | 0.56 |
| has-miR-508-3p (low vs high) | 8.43 (4.51~15.7) | <0·0001 |  | 8.29 (4.39~15.6) | <0·0001 |
