# Supplementary material for: Integrative network biology analysis identifies miR-508-3p as the determinant for the mesenchymal identity and a strong prognostic biomarker of ovarian cancer
Source: Oncogene. 2018 Nov 26;38(13):2305–19. doi: 10.1038/s41388-018-0577-5 (PMC6755993; doi:10.1038/s41388-018-0577-5)
Supplement: Supplementary file 16 — Supplementary Table S7 [file 41388_2018_577_MOESM16_ESM.docx]

| **Supplementary Table S7. GSEA result of has-miR-508-3p expression low vs. high in TCGA data** | | | | |
| --- | --- | --- | --- | --- |
|  |  |  |  |  |
|  |  |  |  |  |
| **Gene Set** | **Enrichment score** | **Normalized enrichment score (NES)** | **p-value** | **FDR** |
| GO_EXTRACELLULAR_STRUCTURE_ORGANIZATION | 0.6791 | 2.3781 | <1e−6 | <1e−6 |
| JECHLINGER_EPITHELIAL_TO_MESENCHYMAL_TRANSITION_UP | 0.7796 | 2.3194 | <1e−6 | <1e−6 |
| GO_COLLAGEN_BINDING | 0.7534 | 2.2397 | <1e−6 | <1e−6 |
| GO_LEUKOCYTE_CELL_CELL_ADHESION | 0.6397 | 2.2233 | <1e−6 | <1e−6 |
| GO_PROTEINACEOUS_EXTRACELLULAR_MATRIX | 0.6253 | 2.2231 | <1e−6 | <1e−6 |
| GO_EXTRACELLULAR_MATRIX | 0.6145 | 2.2097 | <1e−6 | <1e−6 |
| GO_POSITIVE_REGULATION_OF_SMOOTH_MUSCLE_CELL_MIGRATION | 0.8491 | 2.1951 | <1e−6 | <1e−6 |
| GO_REGULATION_OF_SMOOTH_MUSCLE_CELL_MIGRATION | 0.7635 | 2.1665 | <1e−6 | <1e−6 |
| GO_POSITIVE_REGULATION_OF_CELL_ADHESION | 0.6087 | 2.1653 | <1e−6 | <1e−6 |
| GO_POSITIVE_REGULATION_OF_CELL_CELL_ADHESION | 0.6250 | 2.1624 | <1e−6 | <1e−6 |
| GO_EXTRACELLULAR_MATRIX_STRUCTURAL_CONSTITUENT | 0.7156 | 2.1255 | <1e−6 | <1e−6 |
| GO_EXTRACELLULAR_MATRIX_BINDING | 0.7449 | 2.1150 | <1e−6 | 3.73E-06 |
| GO_REGULATION_OF_CELL_CELL_ADHESION | 0.5820 | 2.0844 | <1e−6 | 1.30E-05 |
| GOTZMANN_EPITHELIAL_TO_MESENCHYMAL_TRANSITION_UP | 0.6967 | 2.0780 | <1e−6 | 1.27E-05 |
| REACTOME_EXTRACELLULAR_MATRIX_ORGANIZATION | 0.6773 | 2.0609 | <1e−6 | 4.16E-05 |
| GO_COLLAGEN_FIBRIL_ORGANIZATION | 0.7689 | 2.0543 | <1e−6 | 4.30E-05 |
| GO_EXTRACELLULAR_MATRIX_COMPONENT | 0.6402 | 2.0471 | <1e−6 | 4.99E-05 |
| GO_HEPARIN_BINDING | 0.6136 | 2.0022 | <1e−6 | 1.52E-04 |
| GO_PROTEOGLYCAN_BINDING | 0.7825 | 1.9927 | <1e−6 | 1.86E-04 |
| WU_CELL_MIGRATION | 0.5783 | 1.9608 | <1e−6 | 4.07E-04 |
| GO_INTEGRIN_BINDING | 0.6057 | 1.9205 | <1e−6 | 9.04E-04 |
| GO_FIBRONECTIN_BINDING | 0.7435 | 1.8795 | <1e−6 | 0.0018 |
| REACTOME_COLLAGEN_FORMATION | 0.6544 | 1.8672 | 0.0030 | 0.0022 |
| GO_DENDRITIC_CELL_MIGRATION | 0.8041 | 1.8626 | <1e−6 | 0.0024 |
| GO_CELL_CELL_ADHESION | 0.4957 | 1.8301 | <1e−6 | 0.0040 |
| KEGG_FOCAL_ADHESION | 0.5387 | 1.8245 | <1e−6 | 0.0043 |
| GO_BANDED_COLLAGEN_FIBRIL | 0.8517 | 1.8177 | <1e−6 | 0.0048 |
| VERRECCHIA_EARLY_RESPONSE_TO_TGFB1 | 0.7249 | 2.0887 | 1.32E-05 | 0.004 |
| SARRIO_EPITHELIAL_MESENCHYMAL_TRANSITION_UP | 0.5328 | 1.7605 | 0.0013 | 0.0102 |
| GO_PROTEOGLYCAN_METABOLIC_PROCESS | 0.5916 | 1.7530 | <1e−6 | 0.0111 |
