# Supplementary material for: Integrative network biology analysis identifies miR-508-3p as the determinant for the mesenchymal identity and a strong prognostic biomarker of ovarian cancer
Source: Oncogene. 2018 Nov 26;38(13):2305–19. doi: 10.1038/s41388-018-0577-5 (PMC6755993; doi:10.1038/s41388-018-0577-5)
Supplement: Supplementary file 17 — Supplementary Table S8 [file 41388_2018_577_MOESM17_ESM.docx]

| **Supplementary Table S8. Subtype classification of ovarian cancer cell lines** | | |
| --- | --- | --- |
|  |  |  |
| **Dezső Dataset (GSE50831)** | | |
|  | **posterior probability** | |
| **ID** | **Mesenchymal** | **Non-mesenchymal** |
| SK-OV-3:Ctrl_1 | 0.12 | 0.88 |
| SK-OV-3:Ctrl_2 | 0.09 | 0.91 |
| SK-OV-3:Ctrl_3 | 0.09 | 0.91 |
| TOV-21G:Ctrl_1 | 0.03 | 0.97 |
| TOV-21G:Ctrl_2 | 0.04 | 0.96 |
| TOV-21G:Ctrl_3 | 0.04 | 0.96 |
| OVISE:Ctrl_1 | 0.00 | 1.00 |
| OVISE:Ctrl_2 | 0.00 | 1.00 |
| OVISE:Ctrl_3 | 0.00 | 1.00 |
| TOV-112D:Ctrl_1 | 0.45 | 0.55 |
| TOV-112D:Ctrl_2 | 0.35 | 0.65 |
| TOV-112D:Ctrl_3 | 0.46 | 0.54 |
| OV56:Ctrl_1 | 0.15 | 0.85 |
| OV56:Ctrl_2 | 0.11 | 0.89 |
| OV56:Ctrl_3 | 0.16 | 0.84 |
| COV362:Ctrl_1 | 0.03 | 0.97 |
| COV362:Ctrl_2 | 0.05 | 0.95 |
| COV362:Ctrl_3 | 0.04 | 0.96 |
| COV504:Ctrl_1 | 0.84 | 0.16 |
| COV504:Ctrl_2 | 0.87 | 0.13 |
| COV504:Ctrl_3 | 0.85 | 0.15 |
| COLO-704:Ctrl_1 | 0.02 | 0.98 |
| COLO-704:Ctrl_2 | 0.00 | 1.00 |
| COLO-704:Ctrl_3 | 0.01 | 0.99 |
| COLO 720E:Ctrl_1 | 0.00 | 1.00 |
| COLO 720E:Ctrl_2 | 0.00 | 1.00 |
| COLO 720E:Ctrl_3 | 0.00 | 1.00 |
| A2780:Ctrl_1 | 0.96 | 0.04 |
| A2780:Ctrl_2 | 0.96 | 0.04 |
| A2780:Ctrl_3 | 0.96 | 0.04 |
| COV644:Ctrl_1 | 0.01 | 0.99 |
| COV644:Ctrl_2 | 0.01 | 0.99 |
| COV644:Ctrl_3 | 0.02 | 0.98 |
| EFO-27:Ctrl_1 | 0.54 | 0.46 |
| EFO-27:Ctrl_2 | 0.57 | 0.43 |
| EFO-27:Ctrl_3 | 0.54 | 0.46 |
| OVCAR-3:Ctrl_1 | 0.00 | 1.00 |
| OVCAR-3:Ctrl_2 | 0.00 | 1.00 |
| OVCAR-3:Ctrl_3 | 0.00 | 1.00 |
| OV-90:Ctrl_1 | 0.01 | 0.99 |
| OV-90:Ctrl_2 | 0.01 | 0.99 |
| OV-90:Ctrl_3 | 0.01 | 0.99 |
| KURAMOCHI:Ctrl_1 | 0.11 | 0.89 |
| KURAMOCHI:Ctrl_2 | 0.10 | 0.90 |
| KURAMOCHI:Ctrl_3 | 0.10 | 0.90 |
| OVTOKO:Ctrl_1 | 0.01 | 0.99 |
| OVTOKO:Ctrl_2 | 0.01 | 0.99 |
| OVTOKO:Ctrl_3 | 0.01 | 0.99 |
| OVCAR-4:Ctrl_1 | 0.00 | 1.00 |
| OVCAR-4:Ctrl_2 | 0.00 | 1.00 |
| OVCAR-4:Ctrl_3 | 0.00 | 1.00 |
| OVSAHO:Ctrl_1 | 0.00 | 1.00 |
| OVSAHO:Ctrl_2 | 0.00 | 1.00 |
| OVSAHO:Ctrl_3 | 0.00 | 1.00 |
| EFO-21:Ctrl_1 | 0.02 | 0.98 |
| EFO-21:Ctrl_2 | 0.02 | 0.98 |
| EFO-21:Ctrl_3 | 0.02 | 0.98 |
| COV434:Ctrl_1 | 0.17 | 0.83 |
| COV434:Ctrl_2 | 0.18 | 0.82 |
| COV434:Ctrl_3 | 0.21 | 0.79 |
| CaOv3:Ctrl_1 | 0.00 | 1.00 |
| CaOv3:Ctrl_2 | 0.00 | 1.00 |
| CaOv3:Ctrl_3 | 0.00 | 1.00 |
