# Supplementary material for: Integrative network biology analysis identifies miR-508-3p as the determinant for the mesenchymal identity and a strong prognostic biomarker of ovarian cancer
Source: Oncogene. 2018 Nov 26;38(13):2305–19. doi: 10.1038/s41388-018-0577-5 (PMC6755993; doi:10.1038/s41388-018-0577-5)
Supplement: Supplementary file 18 — Supplementary Table S9 [file 41388_2018_577_MOESM18_ESM.docx]

| **Supplementary Table S9. GSEA result of miR-508-3p inhibitor-treated OvCa cell lines** |  |  |  |
| --- | --- | --- | --- |
|  |  |  |  |
| **Gene Set** | **Enrichment score** | **p-value** | **FDR** |
| SARRIO_EPITHELIAL_MESENCHYMAL_TRANSITION_UP | 0.5070 | <1e−4 | <1e−4 |
| CHARAFE_BREAST_CANCER_LUMINAL_VS_MESENCHYMAL_DN | 0.3783 | <1e−4 | <1e−4 |
| VERHAAK_GLIOBLASTOMA_MESENCHYMAL | 0.3485 | 0.0004 | 0.0050 |
| BOWIE_RESPONSE_TO_EXTRACELLULAR_MATRIX | 0.7982 | <1e−4 | <1e−4 |
| WU_CELL_MIGRATION | 0.3323 | 0.0020 | 0.0190 |
| PID_AVB3_INTEGRIN_PATHWAY | 0.3743 | 0.0246 | 0.1239 |
| KARAKAS_TGFB1_SIGNALING | 0.7163 | <1e−4 | <1e−4 |
| TIAN_TNF_SIGNALING_VIA_NFKB | 0.7217 | <1e−4 | <1e−4 |
| MOSERLE_IFNA_RESPONSE | 0.8523 | <1e−4 | <1e−4 |
| KIM_HYPOXIA | 0.7123 | <1e−4 | <1e−4 |
| FARDIN_HYPOXIA_11 | 0.6513 | <1e−4 | <1e−4 |
| LEONARD_HYPOXIA | 0.6520 | <1e−4 | <1e−4 |
| KRIEG_HYPOXIA_VIA_KDM3A | 0.5484 | <1e−4 | <1e−4 |
| PHONG_TNF_TARGETS_UP | 0.5843 | <1e−4 | <1e−4 |
| SANA_TNF_SIGNALING_UP | 0.6597 | <1e−4 | <1e−4 |
| COULOUARN_TEMPORAL_TGFB1_SIGNATURE_UP | 0.4624 | <1e−4 | <1e−4 |
| CHANDRAN_METASTASIS_UP | 0.3944 | <1e−4 | <1e−4 |
| BIDUS_METASTASIS_UP | 0.4929 | <1e−4 | <1e−4 |
| JAEGER_METASTASIS_UP | 0.6012 | 0.0002 | 0.0027 |
| BECKER_TAMOXIFEN_RESISTANCE_UP | 0.5359 | 0.0002 | 0.0027 |
| HARRIS_HYPOXIA | 0.4677 | 0.0002 | 0.0027 |
| LIAO_METASTASIS | 0.2652 | 0.0028 | 0.0249 |
| COLDREN_GEFITINIB_RESISTANCE_DN | -0.3049 | 0.0032 | 0.0275 |
| CROMER_METASTASIS_UP | 0.4036 | 0.0048 | 0.0381 |
| WANG_METASTASIS_OF_BREAST_CANCER_ESR1_UP | 0.5739 | 0.0076 | 0.0542 |
| CHARAFE_BREAST_CANCER_LUMINAL_VS_MESENCHYMAL_UP | -0.2562 | 0.0098 | 0.0652 |
| CHARAFE_BREAST_CANCER_BASAL_VS_MESENCHYMAL_UP | -0.3455 | 0.0110 | 0.0708 |
| PID_TNF_PATHWAY | 0.4457 | 0.0116 | 0.0735 |
| PEDERSEN_METASTASIS_BY_ERBB2_ISOFORM_7 | 0.2649 | 0.0118 | 0.0744 |
| CROMER_TUMORIGENESIS_DN | -0.4537 | 0.0166 | 0.0954 |
| GO_EXTRACELLULAR_MATRIX_ASSEMBLY | 0.5746 | 0.0306 | 0.1446 |
| GO_REGULATION_OF_CELL_ADHESION | 0.2727 | 0.0014 | 0.0143 |
| GO_SINGLE_ORGANISM_CELL_ADHESION | 0.2852 | 0.0022 | 0.0207 |
| GO_REGULATION_OF_CELL_CELL_ADHESION | 0.2822 | 0.0094 | 0.0635 |
| GO_CELL_ADHESION_MEDIATED_BY_INTEGRIN | 0.6636 | 0.0134 | 0.0809 |
| GO_REGULATION_OF_NEUTROPHIL_MIGRATION | 0.6488 | <1e−4 | <1e−4 |
| GO_REGULATION_OF_SMOOTH_MUSCLE_CELL_MIGRATION | 0.5420 | <1e−4 | <1e−4 |
| GO_POSITIVE_REGULATION_OF_LEUKOCYTE_MIGRATION | 0.4587 | <1e−4 | <1e−4 |
| GO_POSITIVE_REGULATION_OF_NEUTROPHIL_MIGRATION | 0.6555 | 0.0006 | 0.0071 |
| GO_POSITIVE_REGULATION_OF_SMOOTH_MUSCLE_CELL_MIGRATION | 0.5793 | 0.0020 | 0.0190 |
| GO_TISSUE_MIGRATION | 0.3682 | 0.0254 | 0.1268 |
| GO_ANGIOGENESIS | 0.3000 | 0.0036 | 0.0304 |
| GO_EPIDERMAL_GROWTH_FACTOR_RECEPTOR_SIGNALING_PATHWAY | 0.4551 | 0.0038 | 0.0318 |
| GO_BLOOD_VESSEL_MORPHOGENESIS | 0.2782 | 0.0056 | 0.0432 |
| GO_POSITIVE_REGULATION_OF_VASCULAR_ENDOTHELIAL_GROWTH_FACTOR_PRODUCTION | 0.5410 | 0.0080 | 0.0561 |
